# Nanoscale Tracking Combined with Cell-Scale Microrheology Reveals Stepwise Increases in Force Generated by Cancer Cell Protrusions

**DOI:** 10.1021/acs.nanolett.2c01327

**Published:** 2022-08-11

**Authors:** Luka Sikic, Ester Schulman, Anna Kosklin, Aashrith Saraswathibhatla, Ovijit Chaudhuri, Juho Pokki

**Affiliations:** # Department of Mechanical Engineering, Stanford University, Stanford, California 94305, United States; ‡ Department of Electrical Engineering and Automation, Aalto University, Espoo, FI-02150,Finland

**Keywords:** cellular force, nanoscale motion, 3D cell culture, live imaging, microrheology, cancer

## Abstract

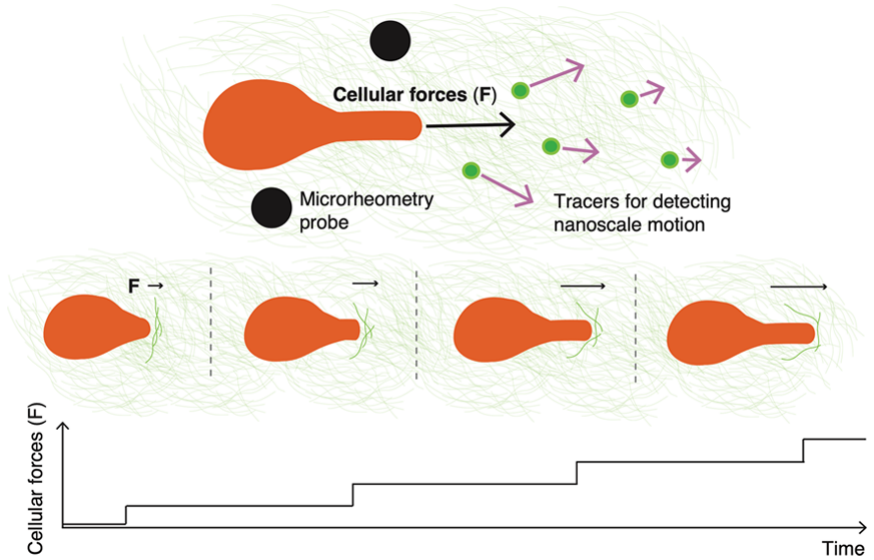

In early breast cancer progression, cancer cells invade through a nanoporous basement membrane (BM) as a first key step toward metastasis. This invasion is thought to be mediated by a combination of proteases, which biochemically degrade BM matrix, and physical forces, which mechanically open up holes in the matrix. To date, techniques that quantify cellular forces of BM invasion in 3D culture have been unavailable. Here, we developed cellular-force measurements for breast cancer cell invasion in 3D culture that combine multiple-particle tracking of force-induced BM-matrix displacements at the nanoscale, and magnetic microrheometry of localized matrix mechanics. We find that cancer-cell protrusions exert forces from picoNewtons up to nanoNewtons during invasion. Strikingly, the protrusions extension involves stepwise increases in force, in steps of 0.2 to 0.5 nN exerted from every 30 s to 6 min. Thus, this technique reveals previously unreported dynamics of force generation by invasive protrusions in cancer cells.

Cells generate forces as part of normal cellular activities,^1−5^ yet, cellular force generation is also a required characteristic of cellular processes involved in diseases^6−8^ such as cancer.^8−12^ In breast cancer, a primary tumor typically develops inside a breast’s mammary duct that is surrounded by a confining basement membrane (BM) (Figure 1). Cancer cells of the tumor must migrate through the extracellular matrix of the BM to invade the neighboring tissue composed of stromal matrix (Figure 1a). To invade further, cancer cells may penetrate the endothelial BM to enter blood circulation and form metastases, tumors at distant sites, the most deadly aspect of breast cancer.^13−15^ The BM is composed of nanoporous matrix that acts as a physical barrier against cancer cell invasion.^16−18^ Since cancer cells are commonly over 10 μm in size, they are unable to fit through the pores of BM matrix without remodeling the matrix. It has been considered that degradative matrix remodeling by cell-secreted enzymes, matrix metalloproteases (MMPs), is the main requirement of invasive migration through the BM, however, pharmaceutical MMP inhibitors in clinical trials have failed to halt breast-cancer progression and metastasis.^19−21^ Recent work has shown that the invasive migration by cancer cells not only involves remodeling by proteases,^22,23^ which biochemically degrade BM but also physical remodeling by cellular forces, which mechanically open up holes in the BM.^8^ This evidence suggests that forces exerted by cancer cells is a clinically relevant metric, providing quantification of the cells’ invasive abilities.

**Figure 1:**
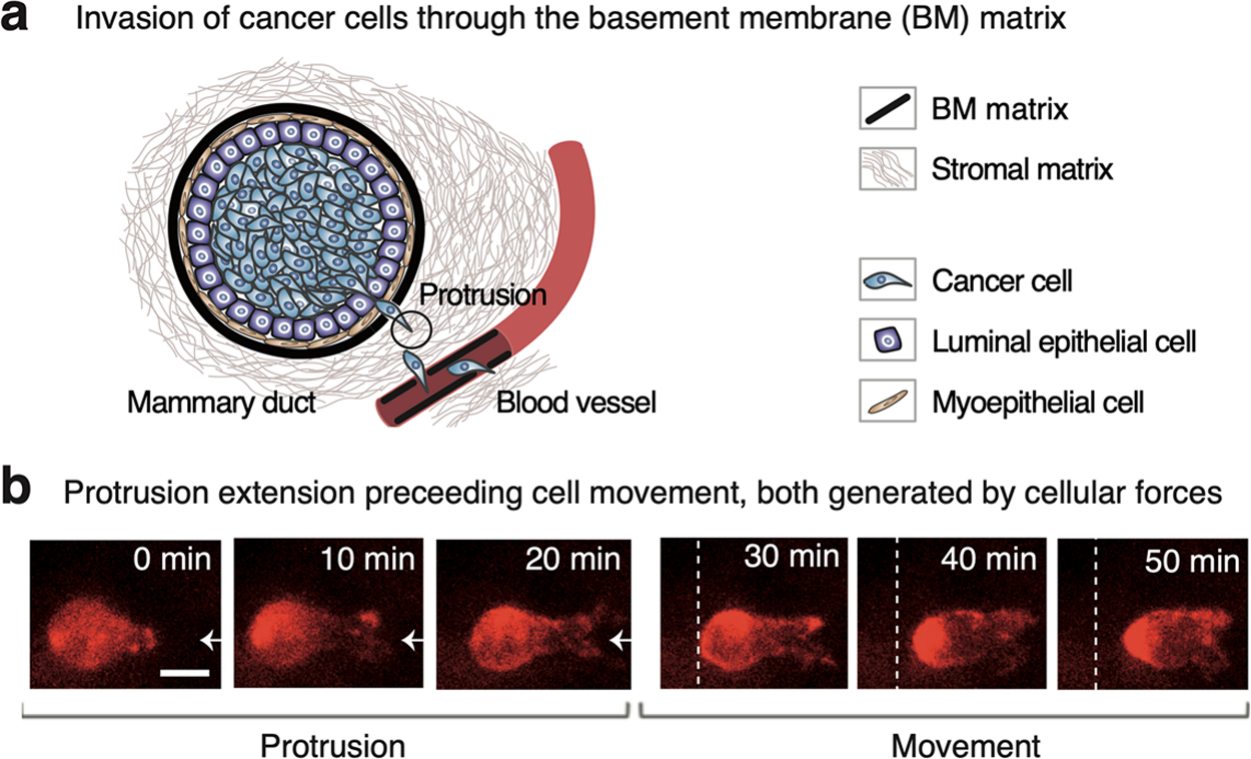
Mammary duct houses a primary tumor and cancer cells that migrate through the BM matrix to invade the neighboring tissue, and these cells extend protrusions to facilitate the migration. (a) Cancer cells penetrate the surrounding BM matrix with the help of cellular protrusions, enabling the cells to enter the stromal matrix. Then, cancer cells similarly invade another BM matrix lining a blood vessel, allowing the cells to access the blood circulation, spread into distant sites and cause deadly metastases. (b) 3D cell cultures of BM-based matrix are used to model the force-generation mechanisms of cancer-cell invasion. A minute-scale extension of a protrusion precedes the migratory cell movement of MDA-MB-231 cancer cells inside the matrix, composed of Matrigel at a concentration of 8.0 mg/mL. Extending a protrusion is indicated with an arrow, and the stationary dashed line illustrates cell movement. The scale bar denotes for 10 μm.

Cancer cells extend invasive protrusions^24^ to exert forces for remodeling nanoporous BM-based matrices^8^ (Figure 1a). This physical remodeling by the protrusions is enabled by complex matrix mechanical properties: viscoelasticity (elastic solid-like and viscous liquid-like characteristics),^25^ and mechanical plasticity (permanent deformation as a response to forces).^8,26,27^ The cancer-cell protrusions exert forces onto the BM-based matrix to permanently deform the matrix enabling the cells to fit through widened pores.^8,28^ Yet, there is a lack of techniques for precise measurements of cellular forces in 3D cell cultures, which are needed to mimic tumor-tissue conditions, since 3D culturing preserves cells’ sensing of the surrounding matrix and relevant genes expression.^29,30^

Current techniques that enable measurements of cellular forces largely focus on 2D culture and involve cells plated on flat synthetic elastic hydrogels (i.e., traction force microscopy) and other rigid two-dimensional substrates (e.g., microfabricated pilar-based measurements).^31−37^ However, these techniques are unable to capture the 3D physical confinement imposed by the tissue surrounding the breast-cancer tumor.^29,30^ Quantifying cellular forces in 3D culture is challenging because of the spatially varying viscoelasticity of the matrix around cells,^10^ arising from heterogeneous distribution of matrix constituents.^25,26,38−41^ Thus, cellular forces in 3D culture can currently be quantified directly only using an average estimate of the matrix elasticity neglecting the local matrix properties,^35^ or indirectly, using force-related displacement fields based on matrix-embedded particles.^10^ Therefore, the mechanisms underlying cancer cells’ force generation in the physiological 3D matrix remain poorly understood.

Microrheology methods enable direct, local measurements of 3D BM-matrix viscoelasticity at the microscale that is the cells’ length scale. Methods based on AFM and optical tweezers (OT) have typically been incapable of measuring the spatially varying properties inside 3D cultures, since they operate at a surface or proximal to the surface^42−44^ (i.e., yet exceptionally OTs measure 500 μm deep^45^). Further, the methods probe one microscale location at the time. Pokki et al.^46^ recently introduced a magnetic-microrheology method using matrix-embedded, 10-μm-diameter magnetic microspheres that simultaneously probe viscoelasticity of multiple microscale locations inside 3D cultures with stiffness levels as in a developing breast cancer.

Here, we developed a technique to precisely quantify cellular forces in 3D culture, generated by breast cancer cells during invasion (Figure 1b). The forces are extracted by combining the measured information on matrix displacements due to the forces, and the local matrix viscoelasticity (Figure 2). This approach was applied to measure forces involved with the extension of protrusions in cancer cells that relates to invasive migration over the BM-based matrix (Figure 1). Live-cell microscopy time lapses of multiple cancer cells were used to obtain data on time-dependent protrusion forces, and the decay of these forces within the matrix (Figures S1 and S2). The forces were first measured at a standard image acquisition time interval of Δt = 15 min, then, at an enhanced temporal resolution of Δt = 6.5–60 s to reveal the dynamics of force generation. Using this technique, we find that the cancer cells exert nanoNewton-scale forces over 15 min, and individual sub-nanoNewton-scale forces are generated over a time scale of seconds, in a step-like manner.

**Figure 2:**
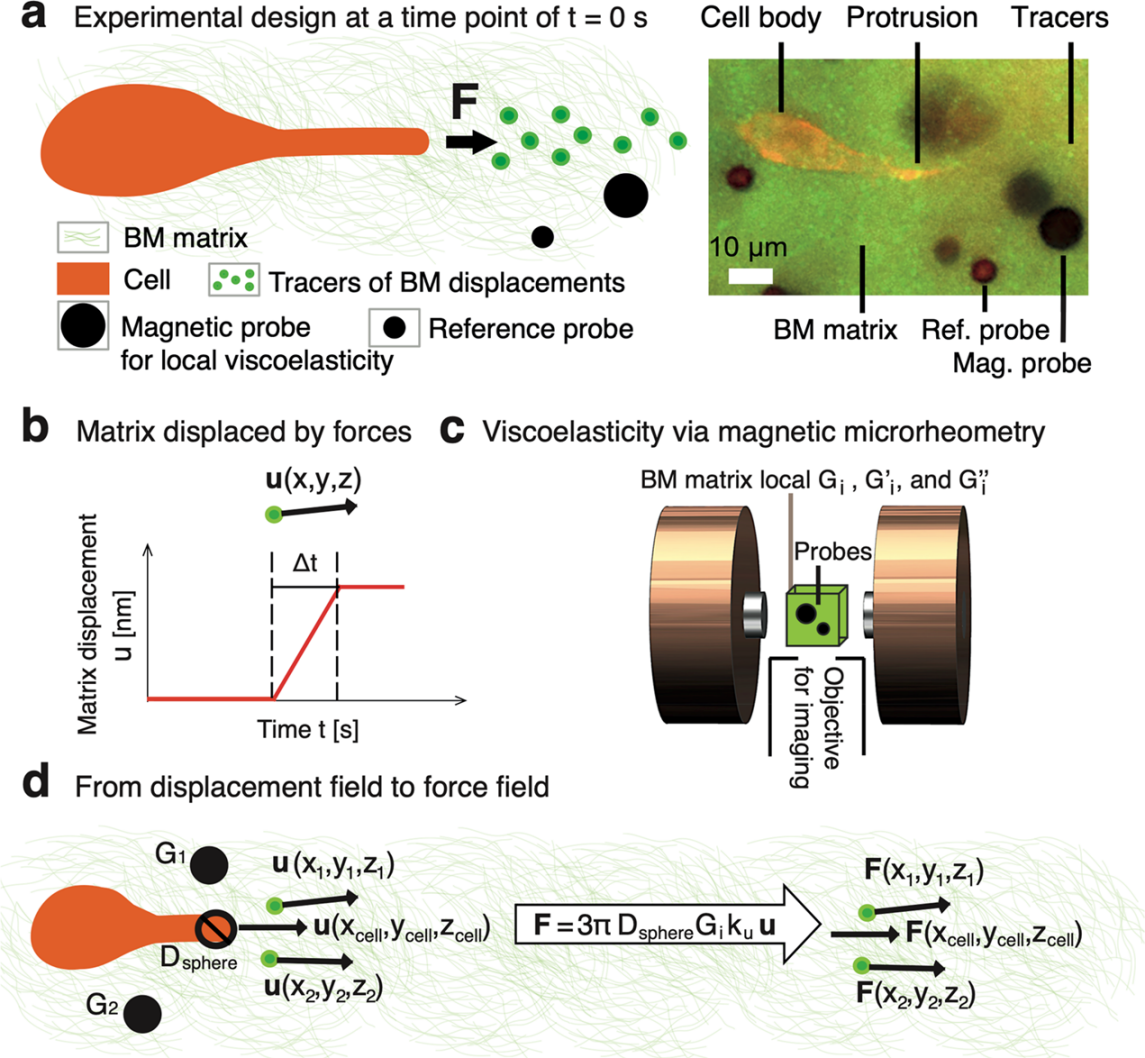
Force generation by cancer-cell protrusions is measured in 3D cell cultures of BM-based matrix by detecting nanoscale displacements and measuring local matrix viscoelastic properties. (a) Forces (**F**) exerted by a cancer-cell protrusion displace nanoscopic parts of reconstituted BM matrix that are detected with 500-nm-diameter green-fluorescent tracers. The matrix displacements are constrained by local matrix viscoelasticity, measured using magnetic and reference probes. (b) Matrix-displacement field (**u**) during a defined time interval (Δt) is used to compute the protrusion forces. An edge-detection algorithm is used to extract the protrusion-tip displacements **u**(x_cell_, y_cell_, z_cell_). Multiple-particle tracking of the tracers is utilized to obtain protrusion extension-related matrix displacements **u**(x, y, z). (c) Microscope-integrated magnetic microrheometer measures microscale matrix viscoelasticity (G_i_, G_i_^′^, and G_i_^″^) by simultaneously actuating 10-μm-diameter magnetic probes and using a microscope camera/objective to image the probe displacement responses. The accurate probe displacement (≤200 nm) is obtained in respect to the sample’s reference-probe position to eliminate environmental noise and vibrations of the microscope objective. Imaging depths within the sample from 50 to 750 μm were used. (d) Force field (**F**) is computed using protrusion-tip diameter (D_sphere_), effective matrix shear modulus (G_i_), displacement field (**u**), and proportional coefficient (k_u_). The k_u_ value is the proportion between time-independent displacements (contributing to the forces) and total displacements, and is determined separately for each time interval (Δt).

## Approach to Measuring Protrusive Forces from Cancer Cells in 3D

The forces generated by cancer-cell protrusions were measured by experimentally detecting the protrusion-tip extensions, tracking matrix displacements, quantifying the local matrix viscoelasticity, and combining the information to compute the forces (Figure 2). The experimental design involved culturing invasive breast-cancer cells (MDA-MB-231) as in the protocol.^8^ The cells were seeded inside a breast-tumor-relevant 3D BM-based matrix, Matrigel at a concentration^47−50^ of 8.0 mg/mL, having a Young’s modulus^38,51,52^ on the order of magnitude of 100 Pa, leading to protrusion formation (Figure 2a). In comparison, healthy breast-tissue cells (MCF-10A), formed no protrusions (Figure S3). The force measurements require adding three types of biocompatible nano/microspheres in the matrix during its preparation. First, yellow–green-fluorescent 500-nm-diameter polystyrene-based spheres served as “tracers” of matrix displacements due to forces. Second, magnetic 10-μm-diameter polystyrene/ironoxide spheres, “magnetic probes”, were used to acquire the local matrix viscoelasticity via magnetic probe-based microrheology. Third, nonmagnetic 6-μm-diameter polystyrene spheres, “reference probes”, were utilized to obtain reference positions for magnetic probes. Further experimental details are in Supporting Information Part B.

Fluorescence microscopy time-lapse imaging was used to detect the displacement field (**u**) involved with the forces exerted by a cancer-cell protrusion tip at a defined time interval (Δt) (Figure 2b). The extension of the protrusion tip was acquired at the red fluorescent channel using edge detection and adapting a custom-made single-object-tracker code by Bergeles^53^ (Video SV1). The positions of the tracers of matrix displacements at the yellow–green fluorescent channel were obtained by applying multiple-particle tracking^54,55^ based on the algorithms of Blair and Dufresne.^56^ These displacements were detected for a maximum time interval of Δt = 15 min that has been previously tested in time-lapse experiments of cancer cells^8^ (Figures S4–S6), and the minimum time interval, Δt = 6.5–10 s, which is closer to the relevant time scale for actin polymerization that is responsible for generating forces.^57^ The displacement resolution is ≅22 nm based on measuring gray scale weighted average at the subpixel resolution using a 30× magnification and an ORCA-Flash-4.0 camera (i.e., a tenth of camera pixel,^58^ corresponding to 0.22 μm).

The matrix displacements due to cellular forces are mediated by the local matrix viscoelastic properties; therefore, our technique uses a microscope-integrated system to measure those properties (Figure 2c). This system, a magnetic microrheometer, and its working principle is described in Pokki et al.^46^ (i.e., system type 2 for stiffer samples^46^). Briefly, a pair of electromagnets is used to generate sinusoidal magnetic forces onto magnetic probes within the matrix with the cells (Equation S1). The applied amplitude^46^ of sinusoidal forces (F̂_magnetic_) displaces the matrix with ≤200 nm, minimizing distraction to the cells, while the applied sinusoidal-force frequency of *f* = 0.05 Hz corresponds to the protrusion-extension dynamics at the time scale of Δt = 1 min (Tables S1–S3). The probe-displacement response to the forces is detected in respect to the displacement amplitude (p̂_magnetic_) and the phase shift (δ) between the forces and the displacements (Equation S2). The F̂_magnetic_, p̂_magnetic_, and δ values are used to extract the following parameters of microscale matrix viscoelasticity: absolute shear modulus, and storage and loss moduli. The absolute shear modulus^59^ of a location i is , where D_probe_ is the magnetic-probe diameter. An effective absolute shear modulus, G_i_ = k_G_G_i_^0^, was used to account for the measured speed values of protrusion extension and subsequent matrix displacement at each respective time scale (Δt). For the purpose, the coefficient k_G_ was determined (Equation S3) to match the effective modulus with the speed-related, frequency-dependent modulus (G_i_^0^) of the matrix (Table S4). Thus, the effective modulus incorporates 0 to 15% higher values than the measured modulus G_i_^0^(*f* = 0.05 Hz) depending on force-generation time scale. The elasticity-related storage modulus is G_i_^′^ = G_i_·cos(δ). The viscous energy dissipation-related loss modulus is G_i_^″^ = G_i_·sin(δ). For calibrating the technique for use with 3D culture of cancer cells, each of the three moduli were investigated during an incubation of 30 and 90 min, and these moduli maintained their values between the incubation times (Table S5). Further, location-dependent differences between the moduli are observed within typical measurement areas of 450·450 μm^2^ (Table S6). The mean ± standard deviation (SD) values of G_i_^0^(*f* = 0.05 Hz) are from 17 ± 2 to 48 ± 17 Pa. The intraexperimental, spatiotemporal viscoelasticity data enables computation of cellular forces.

Forces exerted by each cell’s protrusion tip are computed by combining data about the cell and the surrounding BM-based matrix (Figure 2d). Derived using the definition^59^ of absolute shear modulus, the forces (**F**) exerted by the cell protrusion tip are **F** = 3πD_sphere_G_i_**u′**, where D_sphere_ is the protrusion tip’s characteristic diameter, and **u′** is the effective matrix displacement field. The effective displacements are the displacements that are time independent and relate only to the generated force, specifically, **u′** = k_u_**u**, where the proportional coefficient (k_u_) defines the relation to the measured displacements (**u**). During the time intervals (Δt) of 15 and 1 min, the viscoelastic matrix is expected to undergo material creep,^60^ or increased displacement over time, when a constant force is applied on the matrix. To account for this creep, we conducted finite element method (FEM) simulations based on the rheological properties of the BM-based matrix (Supporting Information Part E, Tables S7–S9, and Figures S7–S12). Based on the simulation, 68.4% and 19.6% of the displacement is due to creep over time scales of Δt = 15 min and Δt = 1 min, respectively (Figure S9). Therefore, we applied proportional coefficients of k_u_ = 31.6% and k_u_ = 80.4% to the measured displacements over time scales of Δt = 15 min and Δt = 1 min, respectively, to determine cell-exerted forces. Further, the measured forces are extracted at the tip of the protrusion at (x_cell_, y_cell_, z_cell_). The decay of these forces within the matrix is calculated based on tracer displacements at different locations (x, y, z). The force decay within the viscoelastic matrix is dominated by elastic properties over viscous-energy dissipation (i.e., the loss factor tan(δ), the ratio between the loss and the storage modulus, is 0.14 ± 0.05 for the matrices with G_i_^0^ = 25.4 ± 8.6 Pa, at a frequency of *f* = 0.05 Hz; 8 matrix samples). Thus, we make the simplifying assumption that the forces decay within the matrix as a function of matrix displacements. The main model of force decay is based on the relationship between force magnitude (F) and protrusion-tip distance (R). In a complementary decay model, local matrix viscoelastic properties were taken into an account (Supporting Information Part G), since the matrix viscoelasticity varies for the locations of individual cells, and the differences in viscoelasticity may impact cellular force generation and force decay within the matrix. Specifically, elasticity (G_i_^′^) in varying orders of magnitude may affect the generation of forces proportionally,^61,62^ and the forces can dampen outward from the cells, inversely proportionally to distance, due to viscous energy dissipation (tan(δ) and G_i_^″^).^63^ The developed technique accounts for spatiotemporally varying viscoelasticity and the dynamic cell–matrix interactions, enabling computation of force generation by cancer cells.

## Cancer-Cell Protrusions Generate NanoNewton-Scale Forces over 15 min

**Figure 3:**
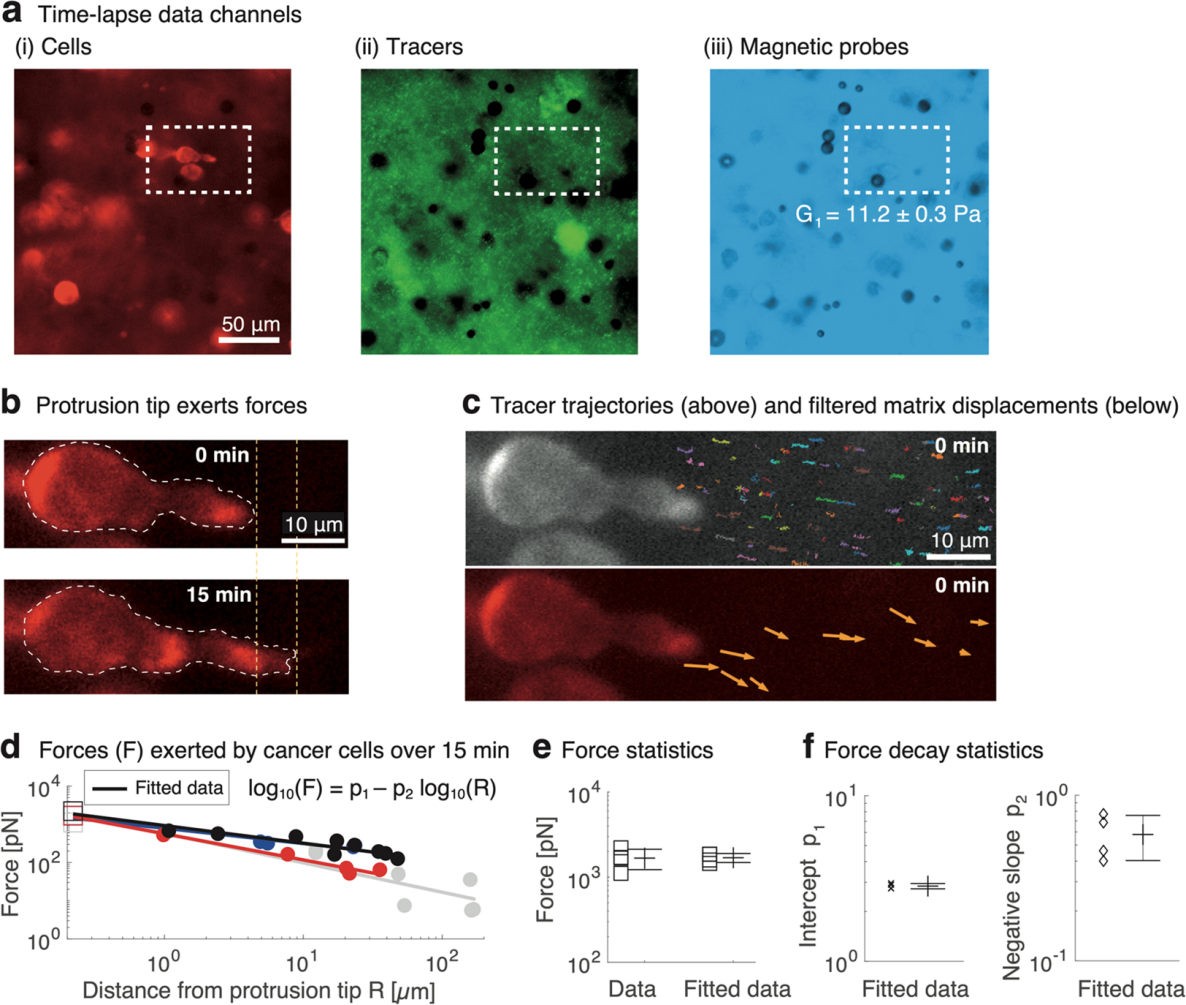
Cancer-cell protrusions exert nanoNewton-scale forces over 15 min that decay outward from the protrusion tip, and the force decay is linear at the logarithmic–logarithmic scale. (a) Time lapses are acquired using three data channels for each cell (fluorescent TRITC), tracers of BM matrix displacements (fluorescent FITC), and magnetic probes for microscale viscoelasticity (bright field). To show the protrusion aligned with x axis, the images are mirrored. (b) Extension of the protrusion tip is measured while the protrusion exerts forces onto the matrix. (c) Colored trajectories show matrix displacements due to protrusion extension based on tracer particles (above). The trajectories are filtered to incorporate the entire used time interval of Δt = 15 min (below). (d) Forces decay with the distance from the protrusion tip for each cancer cell (**Pr < 0.01, Pearson’s test, data points *n* = 7–10/cell, cell count *N* = 4). Squares and filled circles denote forces at the protrusion tip and tracers at different distances from the protrusion tip, respectively. The data from separate cells is denoted by different colors (black, gray, red, and blue). (e) Data for the cell-exerted forces at the protrusion tip that agree between cells (SD/mean <27%). Further, the forces based on this data and fitted data match (n.s. *Pr* > 0.05, two-sided unpaired *t* test, *N* = 4). (f) Force decay outward from the protrusion was consistent for the cells based on the scatter of the intercept (SD/mean <4%) and slope (SD/mean <30%) values for the fit lines (*N* = 4).

Previously, cancer cells have been quantified for their protrusion force-driven migration using a 15 min imaging acquisition interval.^8^ Accordingly, we measured the protrusion forces by recording time lapses using 15 min time intervals (Figure 3) and analyzing the following: protrusion-tip displacement by a cancer cell, matrix displacements, and microscale viscoelasticity (Figure 3a). During a micrometer-scale extension of the protrusion (Figure 3b), the cell-exerted forces are from 1.1 to 2.2 nN, which is within the same order of magnitude as for previously measured 2D cell-exerted forces.^64^ Combining this data with the recorded information on matrix displacements (Figure 3c) enables the computation of decaying forces with distance. In the main decay model, the forces (**F**) show a linear trend outward from the protrusion (R) at the logarithmic–logarithmic scale (Figure 3d), which is confirmed by the FEM simulations for the forces decay over Δt = 15 min (Figure S10). Further, the trend-based forces match with the measured forces at the protrusion-tip edge (Figure 3e), and the decay intercept and slope present a low scatter between the cells (Figure 3f). The complementary decay model was used to incorporate the matrix viscoelasticity into the trend. Testing the combinations of relevant viscoelasticity parameters (Table S10) revealed that incorporating the local loss modulus (G_i_^″^) reduces the data scatter around the trend the most (i.e., R·G_i_^′^ versus **F** data; Figures S13a–c). Further, the matrix is affected by the decaying forces of ≥50 pN as far as 49 μm from the protrusion tip (i.e., an order of magnitude higher forces than thermal forces^65^). The decay also impacts the direction of the forces that become decreasingly aligned with the one of the protrusion extensions with distance (Figure S13d). Cancer-cell protrusions generate forces at the nanoNewton scale over 15 min, and the forces impact within the matrix with a distinct decay trend. To reveal individual forces, shorter time scales need to be considered.

## Individual Sub-NanoNewton-Scale Forces by Cancer-Cell Protrusions Are Generated over a Time Scale of Seconds

**Figure 4:**
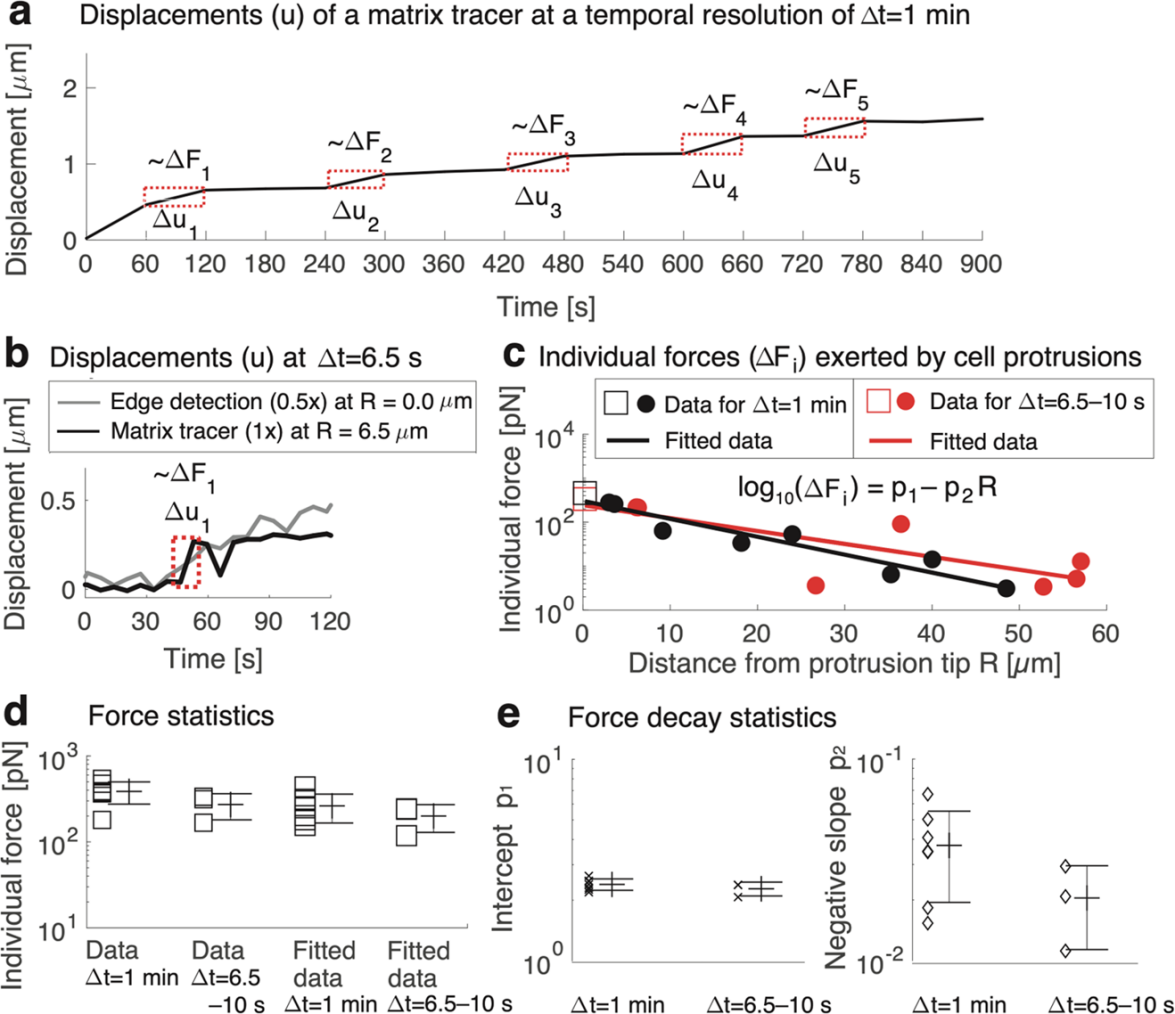
Cancer cells extend protrusions in a stepwise manner on a time scale of 6.5–60 s, with each step exerting a force of 0.2–0.5 nN, decaying linearly with distance at the logarithmic–linear scale. (a) Extension of a cancer-cell protrusion displaces nanoscopic parts of the matrix, measured at a temporal resolution of Δt = 1 min over 15 min. Individual forces (ΔF_i_) follow the dynamics of the displacements of tracers (Δu_i_) and the protrusion tip edge (Figures S9 and S11). Individual force exertions by each cell were detected based on corresponding displacements using the tip edge detection and tracer tracking (red highlight). The shown tracer is 3.1 μm from the protrusion tip. (b) Individual forces associated with the protrusion extension-related displacements (Δu_i_) take place as fast as Δt = 6.5–10 s. The edge-detection data are offset for illustrative purposes. (c) Individual forces decay with distance from the protrusion tip for each cancer cell (*Pr < 0.05 in four cases and **Pr < 0.01 in three cases; Pearson’s test, data points *n* = 7–10 for each cell; cell count *N* = 7). Typical data for cancer cells at Δt = 1 min (*N* = 4) and cancer cells at Δt = 6.5–10 s (*N* = 3) are shown. (d) Measured forces exerted by the protrusions are consistent between the cells (SD/mean < 29% for Δt = 1 min, 2 data points/cell and SD/mean < 34% for Δt = 6.5–10 s, 1 data point/cell). Forces based on the fitted data and the measurements match (n.s. *Pr* > 0.05, two-sided unpaired *t* test between all combinations; *N* = 7, where *N* = 4 for Δt = 1 min, and *N* = 3 for Δt = 6.5–10 s). (e) Force decay was consistent between the cells based on the low scatter values of fit-line intercept (SD/mean is <7% for Δt = 1 min and <8% for Δt = 6.5–10 s) and slope (SD/mean is <48% and <45% for Δt = 6.5–10 s). The intercept and slope values between Δt are insignificantly different (n.s. *Pr* > 0.05, two-sided unpaired *t* test; *N* = 7, where *N* = 4 for Δt = 1 min, and *N* = 3 for Δt = 6.5–10 s).

Cancer-cell protrusions exhibit dynamics on a range of time scales from seconds to minutes, as protrusion extension and migration movement involve fast-acting cytoskeletal polymerization.^24,66^ Therefore, we monitored the force dynamics of the protrusions at an imaging time resolution on the order of seconds to minutes (Figure 4), which has previously been done only in 2D cultures^31^ or microcavities.^67^ At Δt = 1 min imaging intervals, the cancer cells were found to generate forces in two phases that consist of resting phases punctuated by bursts of force exertion (Figure 4a). The results indicate that individual forces by a protrusion are dynamically exerted at the sub-nanoNewton scale over intervals lasting ≥60 s (Figures 4b–c), and the resting phase can take up to 6 min (Table S9). To record the dynamics of individual generated forces, we captured images every 6.5–10 s, and found that the protrusions exert forces from 0.2 to 0.5 nN at the protrusion tip. The force data match between the imaging intervals of Δt = 6.5–10 s and Δt = 1 min (Figure 4c–d). The typicality of the observation (Figure 4b) that only one individual force exertion, lasting for 6.5–10 s, occurs over ≥60 s is supported by the matching force data between the imaging intervals (Figures 4c–e). As an exception, we also found that two subsequent force exertions can be separated by a resting of only 26 s (Video SV2). The measurement and simulation data show that nanometer-scale extensions of cancer-cell protrusions are involved with sub-nanoNewton-scale forces (Figures 4c–d and S11). These extensions/forces relate to comparable strains/stresses (Table S8) that also other cell types exert onto matrices.^68−75^ The main decay model shows that the protrusion forces (**F**) have a linear trend with distance (R) at the logarithmic–linear scale (Figure 4c) that is confirmed by the FEM simulation for both imaging intervals (Δt = 6.5–10 s and Δt = 1 min) (Figure S11). The trend-based forces agree with the measured forces at the protrusion-tip edge (Figure 4d), and the decay intercept and slope show a low scatter between the cells (Figure 4e). The complementary decay-trend model was also used to test the impact of incorporating local matrix viscoelasticity into the trend (Table S11). We found that fitting R·G_i_^″^ with **F** data reduces data scatter around the trend the most (Figures S14a–c and S15a–c). Importantly, the intercept and the slope of the trend match between the time scales Δt = 1 min and Δt = 6.5–10 s, for the main and complementary decay models (Figures 4e, S14c, and S15c). The decaying forces become decreasingly aligned with the direction of protrusion extension with distance (Figures S14d and S15d). The measurements reveal a step-like generation of individual forces from 0.2 to 0.5 nN that decay within the matrix characteristically to the time scale of ≤60 s.

## Discussion and Conclusions

**Figure 5:**
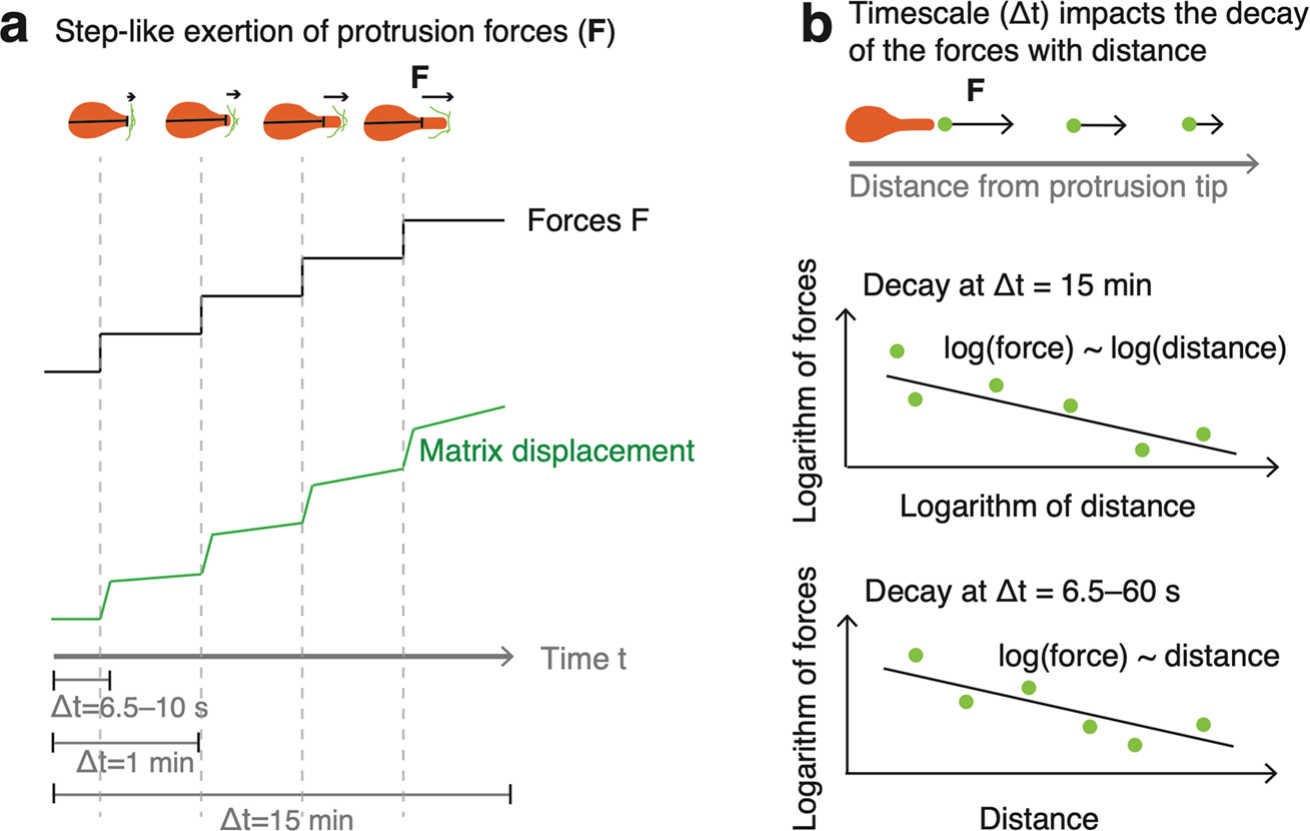
Schematic of cancer cells generating stepwise, sub-nanoNewton-scale increases in force that decays outward from the protrusion. (a) Extension of the cell protrusion displaces the BM-based matrix in a stepwise manner, and measuring the matrix displacements enables calculation of the cell-exerted forces. The forces were analyzed at the time scales of Δt = 15 min, Δt = 1 min, and Δt = 6.5–10 s. The cell body length is indicated by a line crossing the body, while the cell protrusion extends outward and exerts forces on the matrix. (b) Decay of the forces with distance is characteristic to the time scale (Δt). Both force decay behaviors, for Δt = 15 min and Δt = 6.5–60 s, are consistent between experimental measurements and FEM simulations.

We developed a technique for the first time to precisely measure cellular forces during invasion by accounting for the variation in viscoelasticity in 3D culture. The force measurements, which employ magnetic microrheometry, could alternatively use other microrheometry methods (e.g., optical tweezers). This technique, integrated with a fluorescence microscope, was used to explore the recently found mode of cancer-cell migration, where cellular forces applied through invadopodial protrusions drive invasion through nanoporous 3D BM-based matrices.^8^ Besides invasion, protrusive-force generation and pertinent mechanisms relate to intravasation and extravasation by cancer cells.^29,76^ Among the mechanisms, the nucleus has a possible role in driving protrusion-based migration.^77^ The results show that cancer-cell protrusions exert nanoNewton-scale forces over 15 min, comparable to force generation during cell migration in 2D culture,^64^ whereas the previously unknown individual forces narrowed down to the sub-nanoNewton scale. The individual forces were exerted in a stepwise manner (Figure 5a), and this behavior was verified by FEM simulations. These forces decay within the matrix, and the distinct decay trends for the longer (Δt = 15 min) and shorter (Δt = 6.5–60 s) imaging time intervals were captured via experiments and FEM simulation (Figure 5b). Further, the captured data show that protrusive forces are exerted in bursts over 10 s, while the resting periods between two exertions are at the minute scale and, exceptionally, even as short as 26 s; whereas commonly cellular forces have only been evaluated using time scales of minutes. Our data points toward the relevance of the faster force-generation dynamics, due to fast-acting actin polymerization,^24,66^ which is undetectable without significantly faster imaging modes. We detected individual-force generation using enhanced temporal (10 s) and displacement (≅22 nm) resolutions. The results about cancer cells contribute to a better understanding of invasions during early breast-cancer development. This work paves the way for simultaneous 3D culturing and quantification of cell–matrix interactions, with applications in preclinical cancer-drug discovery^11^ and personalized cancer medicine.^78,79^

## Supporting Information Available

Force dynamics/decay, fluorescence images of the cancer cells, MCF10A cells modeling normal mammary epithelium, tracer displacements at two varied distance ranges from protrusion tip, comparison of displacements based on tracers and edge detection, illustrations of the FEM constraints, simulation of force-exertion steps by cells over 1 and 15 min, simulation of the decay of force-caused matrix displacements outward from the protrusion, simulation of force-related stress, force decay modeling incorporating matrix viscoelasticity, mean speed of magnetic-probe displacement and protrusion extension, mean tracer speed at varied distances from the protrusion tip, frequency-dependent viscoelasticity of the Matrigel matrix, viscoelastic moduli within incubation, differences of the absolute moduli between locations, material parameters of the BM-based matrix, simulated cellular force-related stress/strain values at the protrusion tip for varied time scales, force-exertion steps/resting based on protrusion-tip and matrix-tracer speed values, and systematically tested decay models of protrusion forces, definitions of time-dependent magnetic forces onto magnetic probes and the displacement response, and the proportional coefficient to calculate effective shear modulus (PDF)
Edge detection of cancer-cell protrusion edge, imaged every 6.5 s (AVI)
Minimum resting time interval between two force exertions (AVI)

## Supplementary Material

nl2c01327_si_001.pdf

nl2c01327_si_002.avi

nl2c01327_si_003.avi
