# Supplementary material for: Nanoscale Tracking Combined with Cell-Scale Microrheology Reveals Stepwise Increases in Force Generated by Cancer Cell Protrusions
Source: Nano Lett. 2022 Aug 11;22(18):7742–50. doi: 10.1021/acs.nanolett.2c01327 (PMC9523704; doi:10.1021/acs.nanolett.2c01327)
Supplement: Supplementary file 1 — nl2c01327_si_001.pdf [file nl2c01327_si_001.pdf]

SUPPORTING INFORMATION FOR THE ARTICLE:

Nanoscale Tracking Combined with Cell-Scale Microrheology Reveals Stepwise Increases in Force Generated by Cancer Cell Protrusions

Luka Sikic,[¶,‡] *Ester Schulman,*[¶,†] *Anna Kosklin,*[‡] *Aashrith Saraswathibhatla,*[†]
Ovijit Chaudhuri, [*,†] *and Juho Pokki* [*,‡,†]

† Department of Mechanical Engineering, Stanford University, Stanford CA, USA
‡ Department of Electrical Engineering and Automation, Aalto University, Espoo, Finland
¶ Contributed equally to this work
∗ Corresponding authors, with e-mails: chaudhuri@stanford.edu, and juho.pokki@aalto.fi

PART A – GENERAL CONCEPT

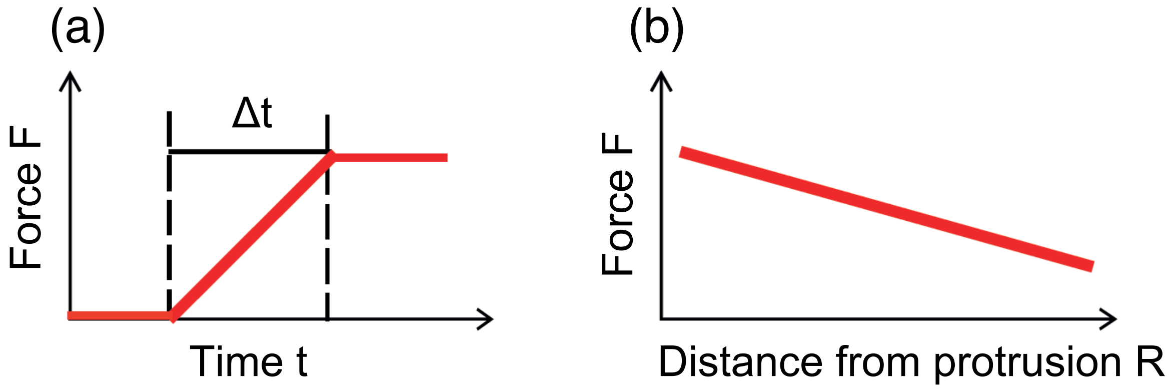

Figure S1. Two types of force information are provided: **(a)** Time-dependent dynamic forces at a constant distance from protrusion tip (R), and **(b)** the decay of forces outward from the protrusion tip during a constant time interval (Δt).

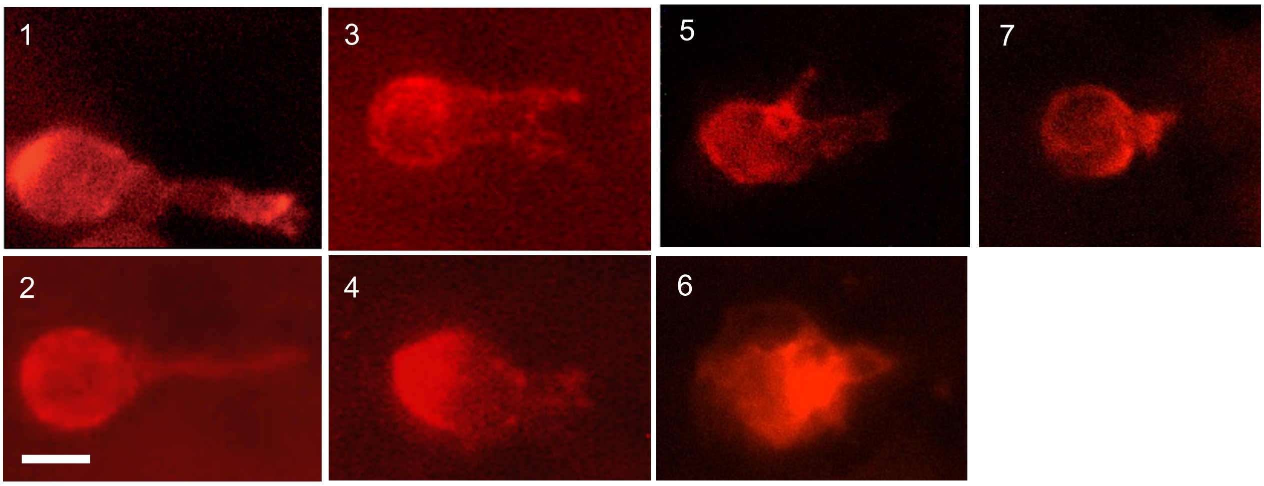

Figure S2. Cancer cells seeded in the BM-based matrix for the force measurements are shown. Each cell is aligned with the x axis, and the scale bar denotes for 10 µm.

PART B – EXPERIMENTAL DETAILS

For each 3D-culture sample, a total volume of 500 μL was initially prepared, which incorporated unpolymerized matrix constituents, cancer cells, and the nano/microspheres.

The unpolymerized matrix constituents included the appropriate volume of Matrigel (Corning #354230) that provides a concentration of 8.0 mg/mL (e.g. 374 μL of a used 10.7 mg/mL product, within a total volume of 500 μL).

Cancer cells were RFP-LifeAct-labeled MDA–MB–231 that were initially 2D cultured as described in the protocols of [1]. Specifically, a cell density of 2.0 million cells/mL was used for each 3D-culture sample. This cell count was determined using a Vi-Cell cell counter (Beckman Coulter). The amount of cells was maintained within the total volume of 500 μL as in the following example: for a 10.7 mg/mL Matrigel product that already fills a volume of 374 μL, the cancer cells were added within a concentrated volume of 61 μL (i.e. containing 1.0 M cells), which enabled to add a further necessary volume of 65 μL containing nano/microspheres.

The three nano/microsphere types used specific dilutions to enable the dispersion of the spheres in the proximity of force-exerting cancer cells. First, the matrix tracers were nanospheres (ThermoFisher FluoSpheres, 500-nm-diameter carboxylate-modified polystyrene spheres, 2wt%) that were diluted with PBS at an optimized ratio of 1:5. This ratio provided a sufficient amount of tracers in the vicinity of the cells, avoiding too dense areas of the tracer nanospheres, and individual tracers were typically distinctly identified. We added a volume of 25 μL of the dilution into the total volume of 500 μL, based on the previous optimization for spherical probes in 3D culture experiments by Pokki et al. [2]. The estimated density of the tracers is $2.5 \cdot 10^9$ tracers/mL. Second, magnetic (Sigma-Aldrich #49664) and reference (Polysciences #15714) probes were both diluted with PBS to reach 0.3wt%, as in Pokki et al. [2] for 3D culture experiments. For each of these two probes, a 20 μL volume of the dilution was used within the total volume of 500 μL. This volume fraction was 20% less than in the earlier optimized protocol in Pokki et al. [2] (i.e. from 25 μL to 20 μL), enabling to further avoid denser areas of the microprobes within the 3D cultures. The estimated density of the magnetic and the reference probes are $2.3 \cdot 10^5$ probes/mL and $1.1 \cdot 10^6$ probes/mL, respectively. All the probes are expected to be physically trapped within the nanoporous [3] Matrigel matrix, and to experience non-covalent interactions only, between each probe's surface (unfunctionalized or carboxylate-functionalized) and the matrix (Matrigel at a concentration of 8.0 mg/mL). No Brownian motion of the probes was observed, which is expected, since the matrix is nanoporous [3], has an increased stiffness (mean$\pm$SD values of the absolute shear modulus are 25.4$\pm$8.6 Pa, 8 samples), and is dominantly elastic (mean$\pm$SD values of the loss tangent are 0.14$\pm$0.05, 8 samples).

These components of each 3D-culture sample were maintained ice cold until polymerization of Matrigel that was performed by incubation for 40 min. Then, the polymerized 3D-culture samples were incubated within a serum-free media for 24 h, as in the previous invasion-study protocol for the same cell line [1] (i.e. FluoroBrite supplemented by GlutaMax and 1% Pen/Strep; Thermo Fisher). Afterward, the serum-free media was aspirated and replaced by media for invasion studies, described in [1] (i.e. FluoroBrite supplemented by GlutaMax, 1% Pen/Strep, 15% FBS, and 50 ng/mL EGF). The time-lapse imaging and force measurements were started after 30 min.

The 3D-culture samples were housed within custom-made polystyrene sample holders, fixed to microscope bottom glass slides using polydimethylsiloxane, having an interior with a width of 3.2 mm, a length of 21 mm, and a height of 12 mm.

A live-cell fluorescence microscope (a customized Nikon Ti2 Eclipse) with a full incubation at 37°C and with a 5% CO_2 supply enabled monitoring of the cancer-cell protrusions and the probes in the experiments. Besides the detected protrusive cells in the Matrigel matrix, multiple cells were less protrusive, round or passive, not exerting forces on the matrix.

For supplementary experiments, we prepared 3D cultures using the MCF10A cell line, a model of normal mammary epithelium [3], [4], within the chosen Matrigel concentration of 8.0 mg/mL. These cells were also seeded at a cell density of 2.0 million cells/mL. The MCF10A cells, expressing RFP-LifeAct, were cultured in Dulbecco's modified Eagle's medium/Nutrient Mixture F-12 (DMEM/F12) medium (Thermo Fisher) supplemented with 5% horse serum (Thermo Fisher), 20 ng/mL EGF (Peprotech), 0.5 μg/mL hydrocortisone (Sigma), 100 ng/mL cholera toxin (Sigma), 10 μg/mL insulin (Sigma), and 1% penicillin/streptomycin (Thermo Fisher). For cell seeding, cells were trypsinized in 0.05% trypsin, and the cell count was determined using the Vi-Cell cell counter. For a desired density of 2.0 million cells/mL, 100 μL of cell solution at a density of 20 million cells/mL was added in an unpolymerized Matrigel (8.9 mg/mL) volume of 898 μL, diluted in high glucose DMEM. The cell–Matrigel solution was incubated right after the seeding. Bright-field and phase-contrast images were collected at time points of 2 h and 6 h after incubation (**Figure S3**).

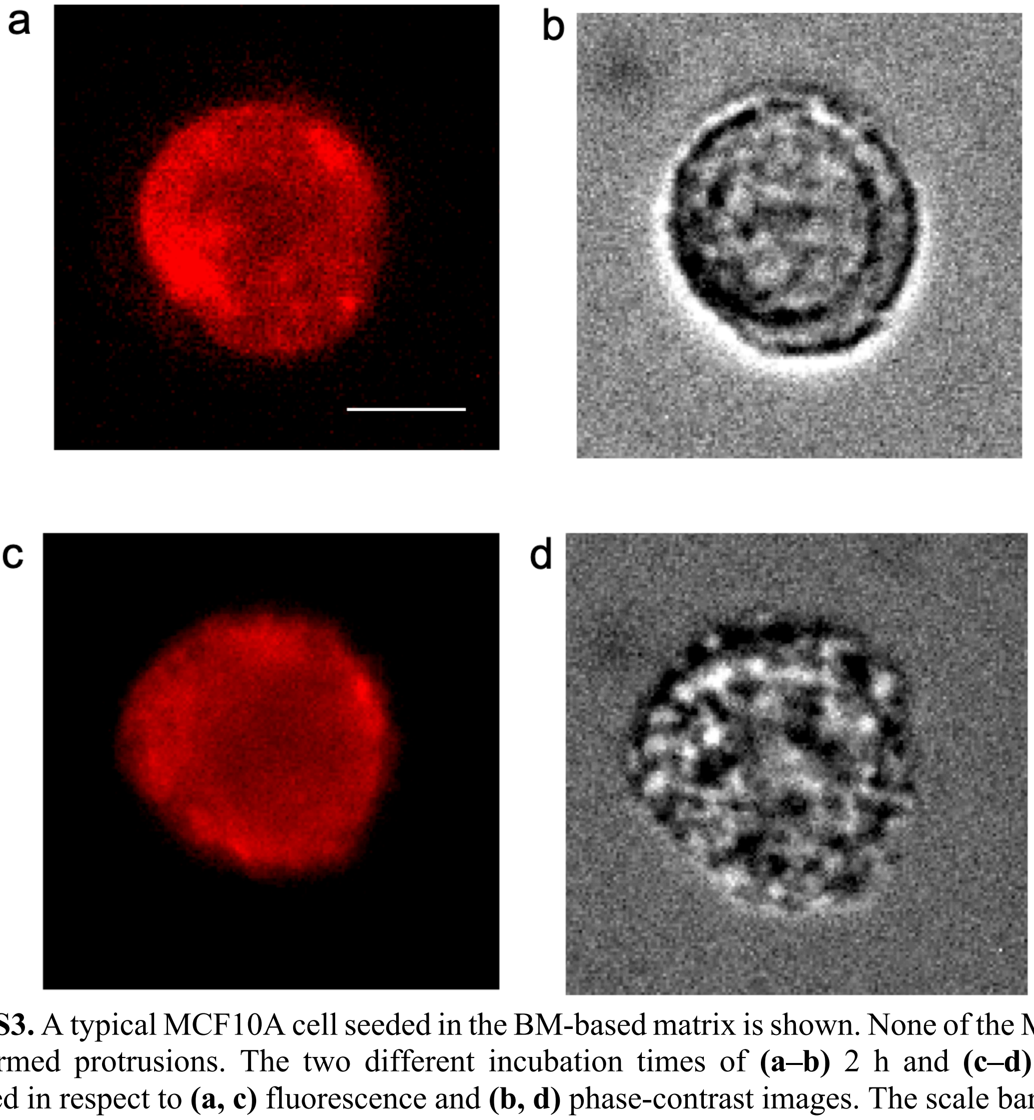

Figure S3. A typical MCF10A cell seeded in the BM-based matrix is shown. None of the MCF10A cells formed protrusions. The two different incubation times of **(a–b)** 2 h and **(c–d)** 6 h are presented in respect to **(a, c)** fluorescence and **(b, d)** phase-contrast images. The scale bar denotes for 10 μm.

PART C – FORCE EXERTION OVER 15 MIN

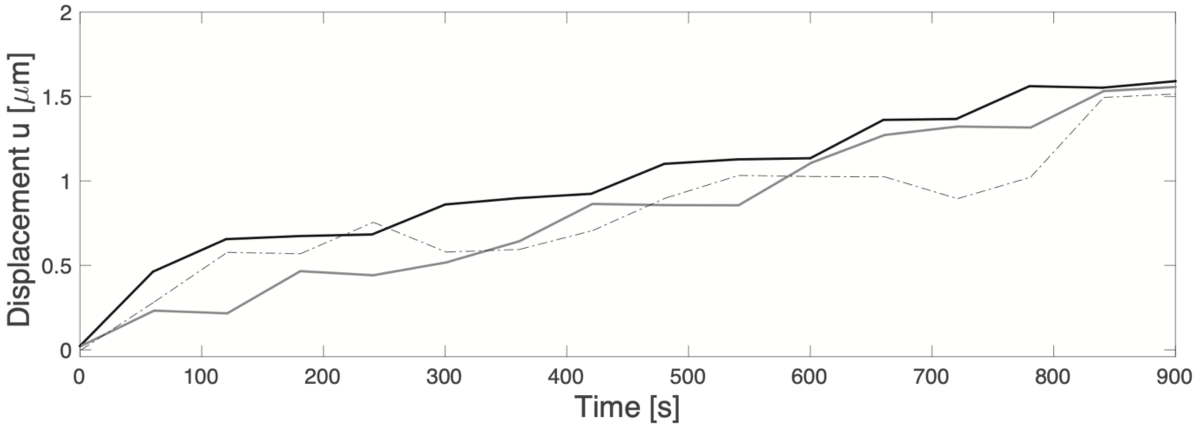

Figure S4. Cell-exerted forces displace the matrix in a time-dependent manner as shown for matrix tracers for three cells. Tracer distances from the protrusion are R < 5 µm. The tracer data for the fourth cell are not shown as the tracers are located further away (R > 10 µm).

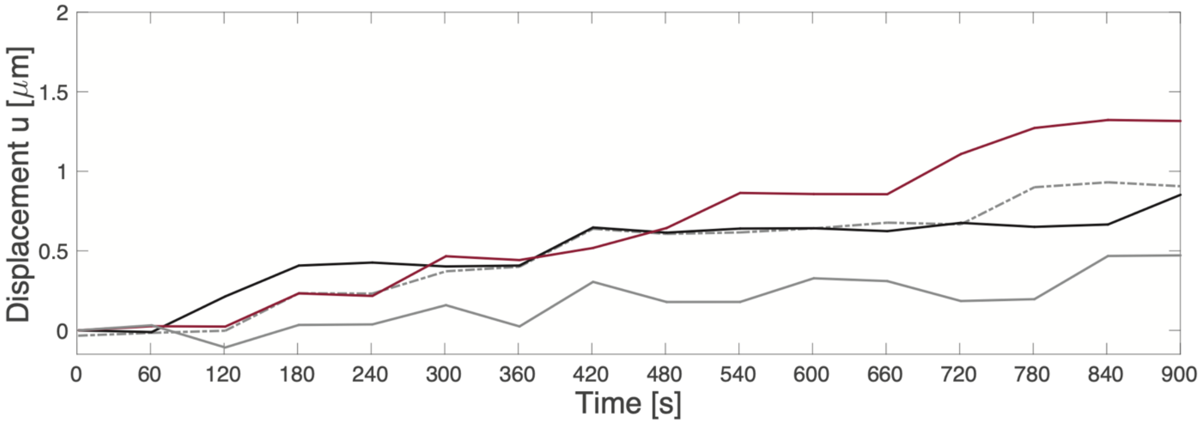

Figure S5. Time-dependent forces displace the matrix similarly further away from the protrusion tip at R = 5.6–12.4 µm. These example tracers are for four cancer cells' force exertions.

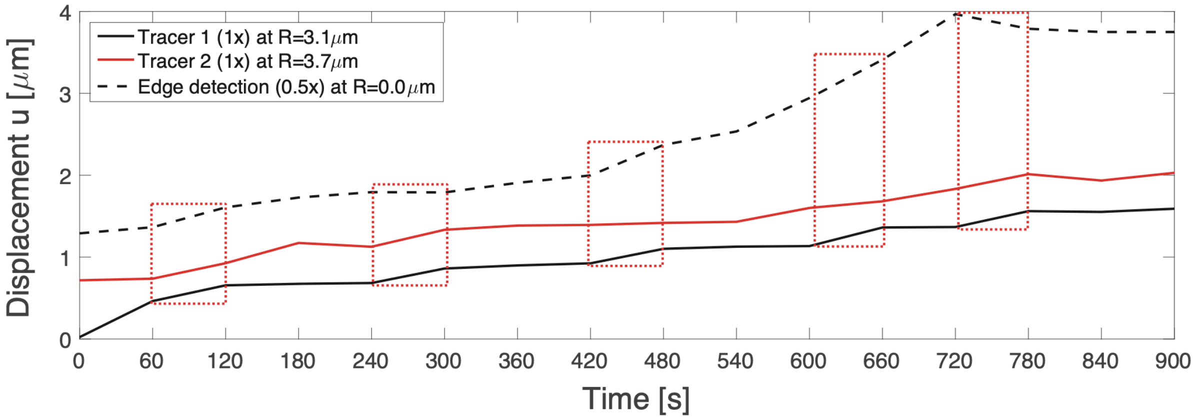

Figure S6. Forces exerted by a cancer-cell protrusion tip displaces the surrounding matrix. The displacement data over 15 min is based on tracking two tracers at the distances of R = 3.1–3.7 µm and detecting the protrusion-tip edge. Force-exertion steps as in Figure 4a are confirmed by consistent displacement data from a minimum of two sources, among the tracers 1–2 and edge detection.

PART D – MICRORHEOMETRY AND FORCE DYNAMICS IN THE BM MATRIX

Time (t) dependent magnetic force ($\mathbf{F}_{\text{magnetic}}$) and the displacement response ($\mathbf{p}_{\text{magnetic}}$) are:

$$\mathbf{F}_{\text{magnetic}} = \hat{\mathbf{F}}_{\text{magnetic}} \cdot \sin(2\pi f \cdot t) \quad \textbf{(S1)}$$

$$\mathbf{p}_{\text{magnetic}} = \hat{\mathbf{p}}_{\text{magnetic}} \cdot \sin(2\pi f \cdot t - \delta) \quad \textbf{(S2)}$$

Table S1. Mean magnetic-probe-displacement speed ($v_{0.05\,\text{Hz}}$) in comparison to mean protrusion-extension speed (v). The $v_{0.05\,\text{Hz}}$ values for varied matrix moduli G_i^0, from mean–SD to mean+SD, are indicated by *. The v values are noted as mean±SD. All values are for the mean characteristic protrusion-tip diameter (D_{sphere}) that has mean±SD values of 4.2±0.9 µm.

Microrheometry at frequency of f=0.05 Hz $v_{0.05\,\text{Hz}}$ [nm/s]	Protrusion extension speed for Δt=15 min v [nm/s]	Protrusion extension speed for Δt=1 min v [nm/s]	Protrusion extension speed for Δt=6.5–10 s v [nm/s]
8.9<br>6.7–13.4 * (N=7)	6.35±1.41<br>(N=4)	8.21±3.13<br>(N=4)	39.98±8.43<br>(N=3)

Table S2. Tracer displacement speed (mean±SD) at a distance of R<5 µm from the protrusion.

Tracer displacement speed for Δt=15 min v [nm/s]	Tracer displacement speed for Δt=1 min v [nm/s]	Tracer displacement speed for Δt=6.5–10 s v [nm/s]
1.64±0.04 (N=3)	4.88±2.45 (N=3)	24.56±5.36 (N=3)

Table S3. Tracer displacement speed of Δt=15 min for varied R, with mean±SD values noted.

R=5–14 µm v [nm/s]	R=15–29 µm v [nm/s]	R > 30 µm v [nm/s]
1.00±0.47 (N=3)	0.44±0.34 (N=2)	0.25±0.15 (N=3)

$$k_G = G_i^0\left(f = \frac{v(\Delta t)}{v_{0.05\,\text{Hz}}} \cdot 0.05\,Hz\right) \cdot \frac{1}{G_i^0(f = 0.05\,Hz)} \quad \textbf{(S3)}$$

Table S4. Frequency-dependent viscoelasticity of Matrigel at a concentration of 8.0 mg/mL. At least 3 biological replicates were used for these measurements.

Frequency f [Hz]	Absolute shear modulus $G_i^0 = \lvert G_i^{0\prime} + i\,G_i^{0\prime\prime} \rvert$ [Pa]	Storage shear modulus $G_i^{0\prime}$ [Pa]	Loss shear modulus $G_i^{0\prime\prime}$ [Pa]
0.022	65.61±12.66	65.18±12.57	7.47±1.50
0.05	65.20±13.32	64.87±13.24	6.55±1.40
0.10	67.82±13.86	67.48±13.80	6.85±1.32
0.22	71.08±14.44	70.75±14.35	6.84±1.64
0.46	75.03±15.39	74.73±15.30	6.67±1.68
1.0	82.80±17.57	82.33±17.28	8.81±3.13

Table S5. Absolute shear modulus (G_i^0) and phase shift (δ) during an incubation of 30 min and 90 min. The absolute shear modulus, and storage and loss moduli change insignificantly between the incubation times (n.s. Pr<0.05, two-sided unpaired t-test; probe data within a typical sample).

Probe number (repetition number)	G_i^0 after 30 min [Pa]	G_i^0 after 90 min [Pa]	δ after 30 min [ ]	δ after 90 min [ ]
1 (1)	45.98	46.60	0.0713	0.1781
1 (2)	52.37	47.70	0.1440	0.0490
2 (1)	42.17	40.82	0.0340	0.1371
2 (2)	45.39	40.01	0.1460	0.1184

Table S6. Repetitive measurements of the absolute modulus (G_i^0) for eight distinct probed, microscale locations I–VIII with an increasing mean value. A significant difference between the locations I (minimum mean value) and VIII (maximum mean value) is found (*Pr<0.05, two-sided unpaired t-test; probe data within a typical sample).

G_i^0 in I [Pa]	G_i^0 in II [Pa]	G_i^0 in III [Pa]	G_i^0 in IV [Pa]	G_i^0 in V [Pa]	G_i^0 in VI [Pa]	G_i^0 in VII [Pa]	G_i^0 in VIII [Pa]
18.27	24.73	21.79	20.60	12.94	13.61	48.74	54.74
15.16	14.06	16.16	15.55	16.41	53.01	22.04	27.71
18.49	18.85	19.02	32.16	30.33	25.75	N/A	60.18

PART E – FINITE ELEMENT MODELING (FEM) OF FORCE EXERTIONS WITHIN THE BM-BASED MATRIX

Table S7. Material parameters of the BM-based, Matrigel matrix. Incompressibility (Poisson's ratio ν=0.5) was assumed within the accuracy enabled by the FEM modeling (i.e. ν=0.49 was used). A linear relation between the relaxation time definitions is assumed (i.e. $\tau_{1/2}$ and τ). The initial yield stress was considered negligible, thus set to a minimal, non-zero value.

Parameter	Name	Magnitude	Unit	Justification
G_i^0	Shear modulus	25.4±8.6 (8 samples)	Pa	Experimental value
ν	Poisson's ratio	0.49	[ ]	Incompressibility
E_i^0	Young's modulus	$\frac{G_i^0}{2(1+\nu)}$	Pa	Standard equation
$\tau_{1/2}$	Relaxation half time	≅70	s	Reference [5]
τ	Relaxation time	$\tau_{1/2} \cdot \frac{1/2}{1/e}$	s	Conversion
η	Damping constant	$G_i^0 \cdot \tau$	Pa · s	Standard equation
σ_{y0}	Initial yield stress	Minimal; 20	Pa	Reference [6]
ρ	Density	≅1000	$\frac{kg}{m^3}$	Assumption

Comsol Multiphysics version 5.6 was used for the FEM simulation of the interaction between the individual cell-exerted forces and the micromechanical responses of the BM-based, experimental Matrigel matrix. A built-in constitutive model based on standard linear solid element in series with Perzyna viscoplasticity without hardening was chosen due to its descriptivity of a variety of cell-culture materials [6]. Small deformations and geometric linearity of the matrix was assumed. The total strain (ε) was computed using additive strain decomposition of the elastic ($\varepsilon_{\text{elastic}}$), viscoelastic ($\varepsilon_{\text{viscoelastic}}$) and viscoplastic ($\varepsilon_{\text{viscoplastic}}$) strains: $\varepsilon = \varepsilon_{\text{elastic}} + \varepsilon_{\text{viscoelastic}} + \varepsilon_{\text{viscoplastic}}$. The elastic strain is proportional to forces, the viscoelastic strain incorporates creep and stress relaxation effects of the matrix, and the viscoplastic strain accounts for permanent matrix deformation. The simulation incorporates the matrix's material parameters, including its viscoelastic and -plastic properties (Table S7).

Due to the symmetry of the protrusion, we performed a 2D axisymmetric simulation about the forces exerted by the cancer-cell protrusion tip. The geometrical dimensions of the simulation, illustrated in Figures S6–S7, consisted of a square with an edge length of 112.5 μm, with the left vertical boundary being the symmetry axis and a symmetry constraint. The edge length was determined by the experimental data in which no displacements had been measured beyond 112.5 μm. These conditions were considered to hold the spatial domain Ω in the BM-based matrix, with appropriate boundary and initial conditions (Figure S6–S7). As two possible scenarios, we accounted for a force- and displacement-controlled system of force exertion by the protrusion.

First, the force-controlled system involved a simulation of the displacement field (output) caused by dynamically exerted individual force values (input). These input force values are based on the experimental measurements and analytical calculations (i.e. an mean individual force of 272 pN, measured Δt=6.5–10 s, was used). Numerical results were obtained by solving the model with the imposed displacement and traction boundary conditions (Figure S7). Specifically, we applied a uniformly distributed boundary load having the input force in the z direction of the lower horizontal boundary, towards the viscoelastic/plastic solid (i.e. a portion $\partial_2\Omega\colon \mathrm{z} = 0, \mathrm{r} \in [0, 2\mu m]$.

The matrix displacements undergo insignificant creep displacements (≤5.5% of total displacements) as a response to the individual forces over an interval of Δt=6.5–10 s (Figures S8a–b), since this interval is an order of magnitude shorter than the viscoelastic timescale of the matrix [5]. NB: The relaxation half time $\tau_{1/2}$, or the stress half time in stress relaxation, is indicated in Table S7. For measuring the forces over a timescale of Δt=1 min, the creep displacements are 19.6% of total displacements, assuming only one or two force exertion steps that were detected

(Figures S8b–d). For measuring the forces over a timescale of Δt=15 min, the creep displacements are 68.4% of total displacements, assuming a mean value in the case of detected four or five force exertion steps (Figures S8e–h). Possible creep displacements from three to six force exertion steps are shown. The simulated displacement field matches with the measured displacement field for all the timescales (Figures S9–S10). These simulated displacement results, verified by experiments, were used as an input to the displacement-controlled system.

In the second scenario, we assumed a displacement-controlled system in which we used a prescribed displacement boundary condition (input) estimating the time-dependent displacements of the boundary (Figures S6–S7). Thus, the simulation was adjusted to solve for the force-related stress (output) caused by the input displacements along z axis (Figure S11).

The FEM results for the force- and displacement controlled systems are summarized in Table S9.

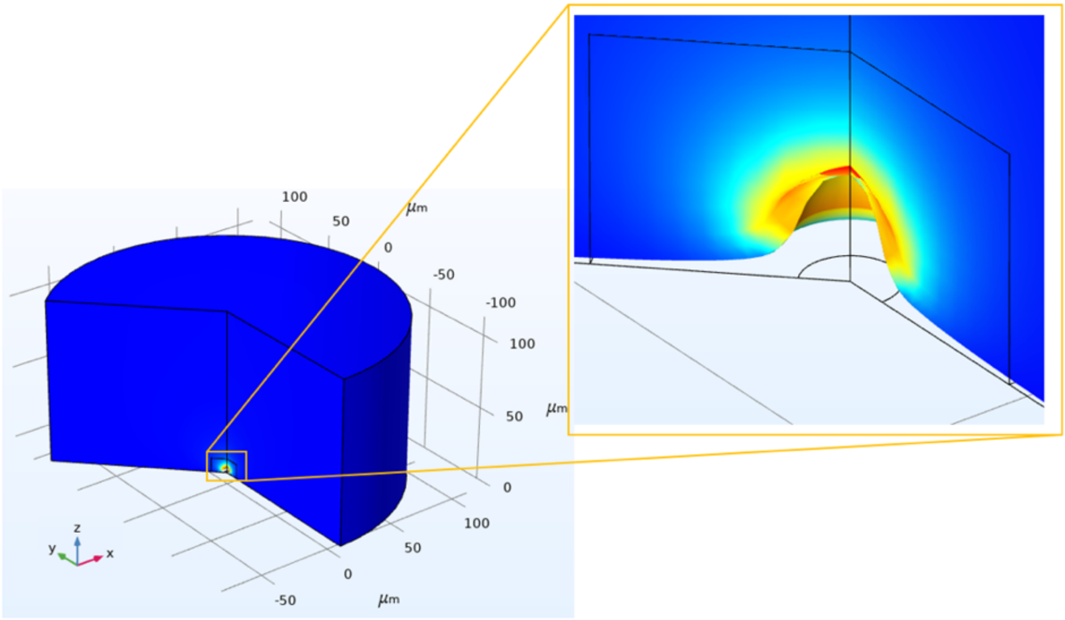

Figure S7. 3D modeling through the 2D axisymmetric Comsol-based FEM simulation. The zoomed area shows how the BM-based matrix is displaced as the cancer-cell protrusion extends. The colors correspond to the matrix stresses.

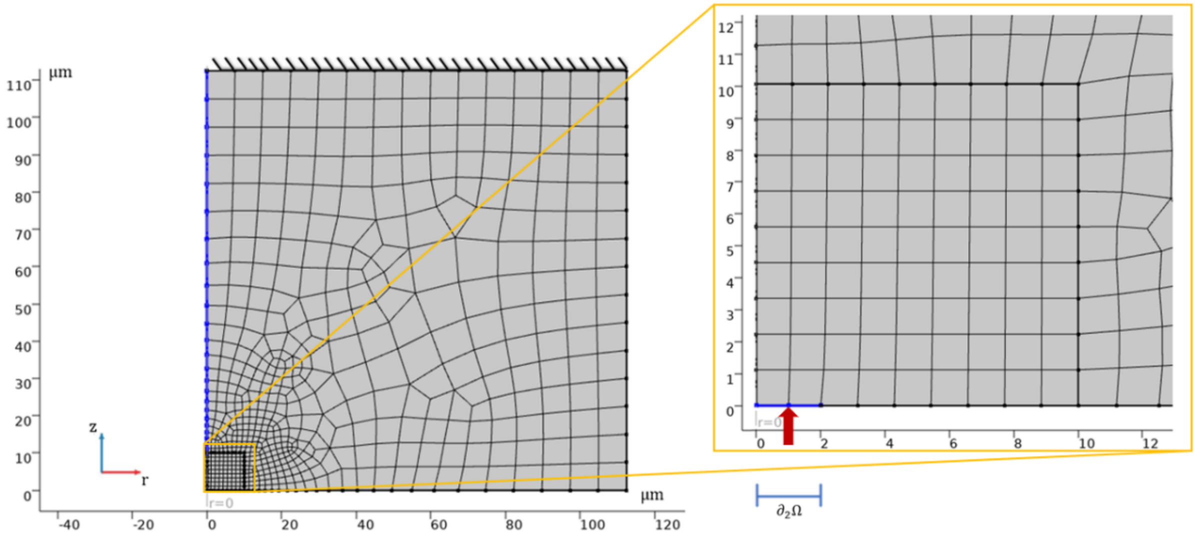

Figure S8. 2D axisymmetric FEM model, optimized mesh and boundary conditions. The zoomed area shows the area of the finest mesh. The symmetry axis is the vertical z-axis (in blue) and a Dirichlet boundary condition is applied on the upper horizontal boundary. A boundary load is applied on a portion $\partial_2\Omega$ of the boundary (in blue in the zoomed area) in the direction of the boundary normal and towards the viscoelastic/plastic solid (red arrow).

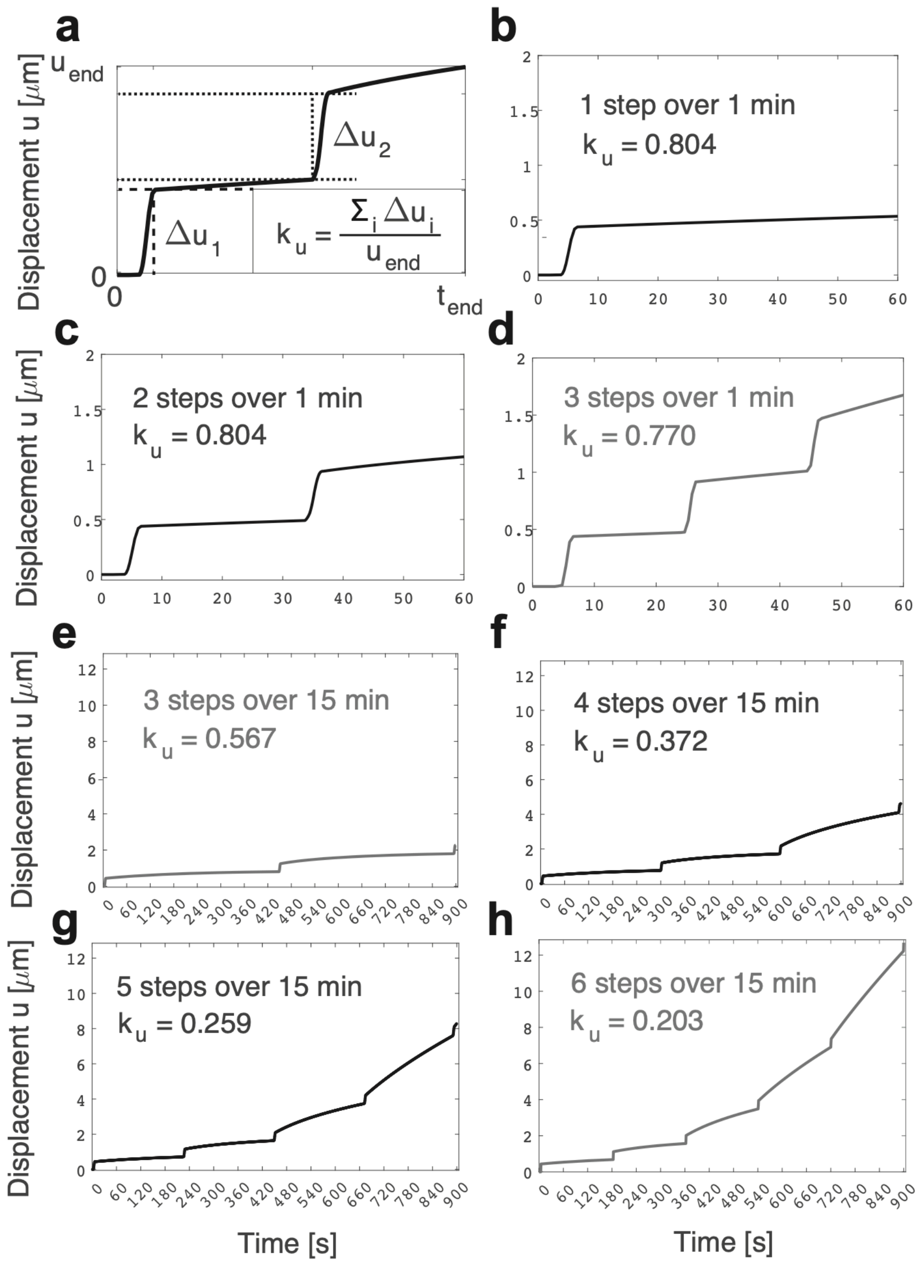

Figure S9. Simulated matrix displacements using a varied number of force-exertion steps. The matching of displacements between this simulation and experiments is shown in Figures S9–S10. **(a)** Principle of calculating the k_u coefficient is presented. An individual force exerted over Δt=6.5–10 s has a k_u value of 0.945–0.963. **(b–d)** A number of 1–2 force-exertion steps provide an equal k_u coefficient of 0.804 during Δt=1 min. For 3 force-exertion steps, the k_u coefficient is 0.770 **(e–h)** Simulation data for a number of 3–6 force exertion steps over Δt=15 min. The measurement data involved a number of 4–5 analogous steps over 15 min, denoting a k_u value from 0.259 to 0.372 (i.e. mean±SD is 0.316±0.080). Each simulated force-exertion step of 272 pN is separated by equal resting times, depending on the number of steps. The number of force-exertion steps in the black plots have been confirmed to happen, yet, also the step number in the grey plots may happen.

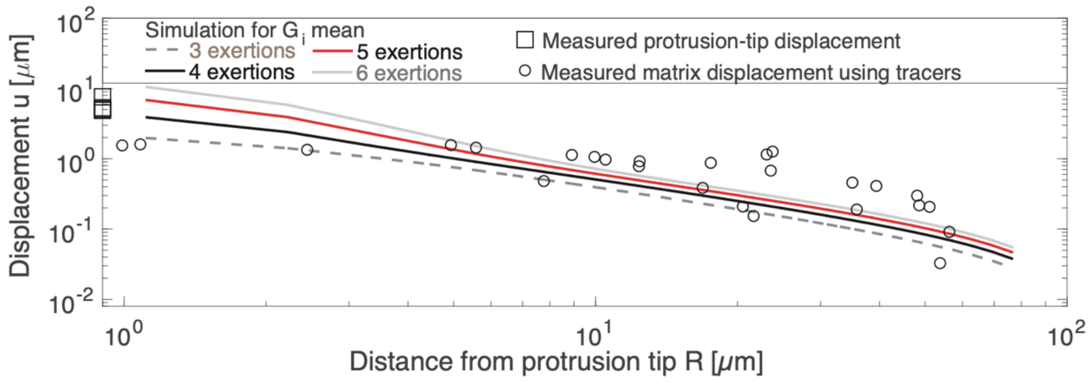

Figure S10. Simulated and measured displacements are aligned over Δt=15 min. A particular matching is shown for 4–5 force-exertion steps, detected in measurements.

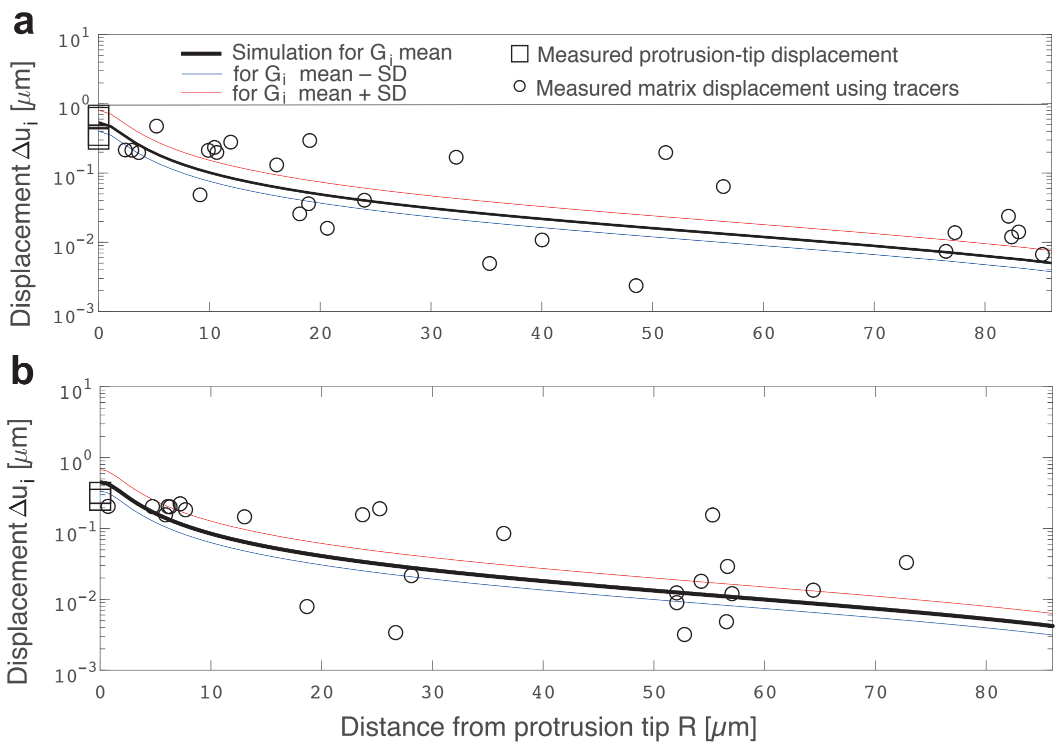

Figure S11. Simulated and measured displacements match for both timescales of (a) Δt=1 min and (b) Δt=6.5–10 s.

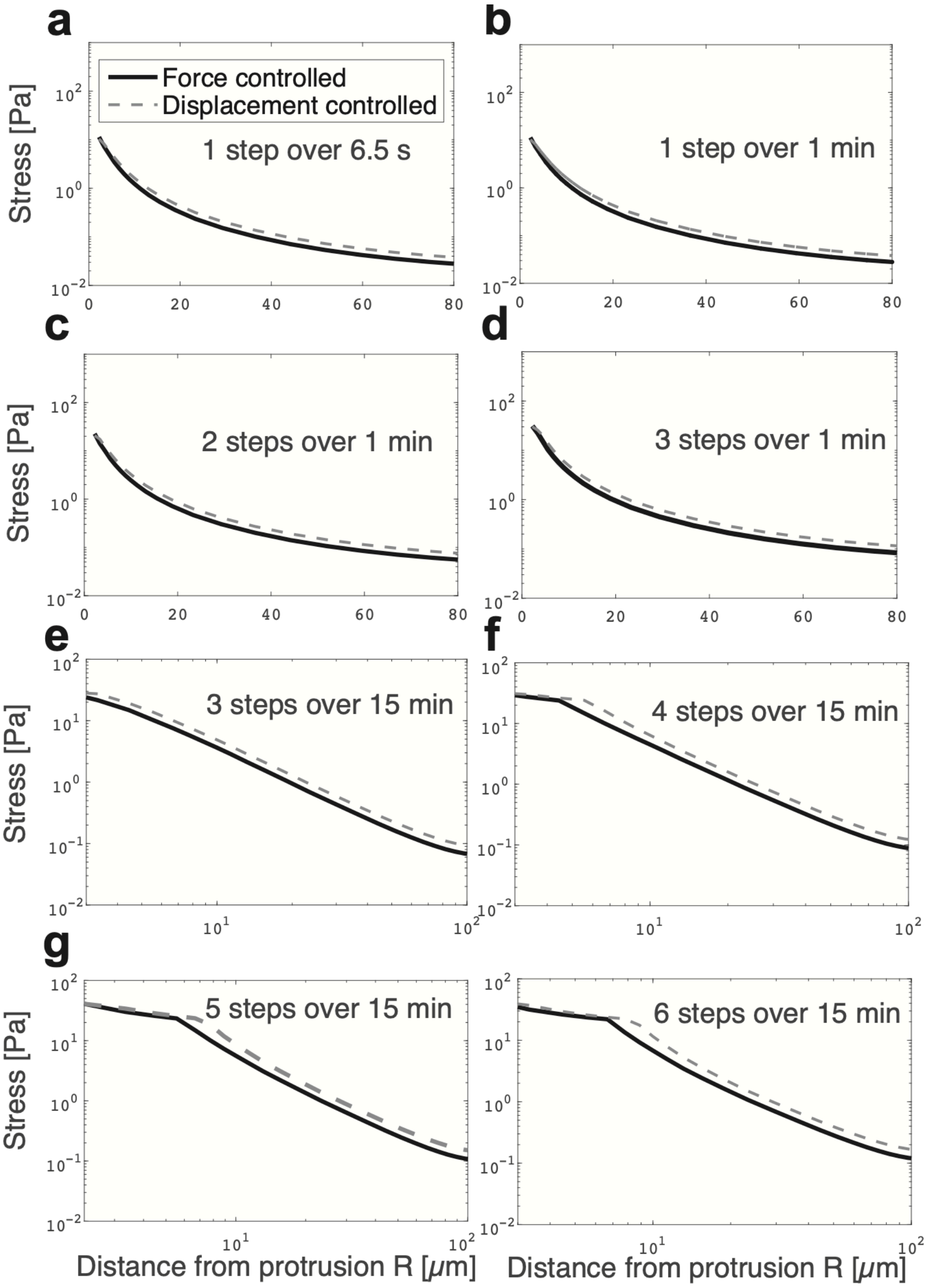

Figure S12. Simulation results of force-related stress as a function of distance from protrusion, for the force- and displacement-controlled protrusion extension. The simulation provides consistent results for both cases.

Table S8. Simulated cellular force-related stress, strain and displacement values at the protrusion tip for the varied timescales and force-exertion step numbers. (**a**) Force-controlled and (**b**) strain-controlled protrusion extension is assumed.

Timescale (steps) / Parameter	Δt=6.5-10 s	Δt =1 min			Δt =15 min			
	(1)	(1)	(2)	(3)	(3)	(4)	(5)	(6)
a) Force [nN]	0.27	0.27	0.54	0.82	0.82	1.09	1.36	1.63
a) Stress [Pa]	11.37	11.37	22.63	31.19	32.18	34.35	40.81	46.04
a) Displacement [μm]	0.45	0.54	1.06	1.69	2.28	4.63	8.22	12.57
a) Normal strain [ ]	0.075	0.090	0.176	0.258	0.359	0.585	0.987	1.418
b) Force [nN]	0.27	0.27	0.54	0.82	0.82	1.09	1.36	1.63
b) Stress [Pa]	10.37	10.12	20.75	30.12	30.14	34.11	40.90	45.95
b) Displacement [μm]	0.45	0.54	1.06	1.69	2.28	4.63	8.22	12.57
b) Normal strain [ ]	0.068	0.080	0.161	0.249	0.336	0.580	0.990	1.415

PART F – ANALYSIS OF STEPWISE EXERTION OF FORCES

Table S9. Mean matrix displacement speed (v) between subsequent frames that were imaged every 1 min for a duration of 15 min. The data of the matrix tracers at R < 5 µm and the protrusion tip for three cancer cells are shown, while the data for the fourth cell are not shown, as the tracers are further (R > 10 µm). To define whether a force-exertion step exists, a threashold is used. For the data of each cell, the threashold is considered for three speed values: the ones of two tracers, and the one of the protrusion tip (i.e. a 75% of the protrusion-tip speed is used to be able to compare the tip speed value to the decayed speed values of tracers; Figure S10). If the tracer speed values are at least the threashold of 0.15 µm/min, a potential step is defined to exist. If the value is below the threashold, a potential rest is defined. A step or a rest in force exertion is assumed to exist, if a minimum of two speed values indicate a (potential) step or a (potential) rest, respectively. For subsequent steps, we consider a range, since a force-exertion step may happen between the captured frames, or in the middle of the frames. Based on this computation, the cells 1, 2 and 3 may have 5–7, 4–6 and 3–4 steps, respectively. All the cells may have 4–5 steps.

Data \ Frames	1-2	2-3	3-4	4-5	5-6	6-7	7-8	8-9	9-10	10-11	11-12	12-13	13-14	14-15
**Cell 1** Tracer 1 v [nm/min]	20	189	247	-46	207	52	8	25	14	169	81	151	180	-8
status	rest	step	step	rest	step	rest	rest	rest	rest	step	rest	step	step	rest
Tracer 2 v [nm/min]	440	192	18	10	177	40	26	177	27	7	227	6	194	-9
status	step	step	rest	rest	step	rest	rest	step	rest	rest	step	rest	step	rest
Tip of protrusion v [nm/min]	124	514	95	98	-40	120	402	473	335	870	568	585	5	-220
status	rest	step	rest	rest	rest	rest	step	step	step	step	step	step	rest	rest
**Combined status**	**rest**	**1 step**	**rest**		**1 step**	**rest**		**1 step**	**rest**	**2-4 steps**				**rest**
**Cell 2** Tracer 1 v [nm/min]	192	65	88	194	42	215	230	0	254	10	143	26	5	-7
status	step	rest	rest	step	rest	step	step	rest	step	rest	rest	rest	rest	rest
Tracer 2 v [nm/min]	209	-16	250	-24	76	125	220	-7	-1	252	164	50	-6	216
status	step	rest	step	rest	rest	step	step	rest	rest	step	step	rest	rest	step
Tip of protrusion v [nm/min]	331	-156	-163	-219	94	446	494	129	385	371	100	874	-48	652
status	step	rest	rest	rest	rest	step	step	rest	step	step	rest	step	rest	step
**Combined status**	**1 step**	**rest**				**1-2 steps**		**rest**	**1-2 steps**		**rest**			**1 step**
**Cell 3** Tracer 1 v [nm/min]	284	294	-9	187	-177	16	111	193	134	-6	-2	-129	-127	472
status	step	step	rest	step	rest	rest	rest	step	rest	rest	rest	rest	rest	step
Tracer 2 v [nm/min]	32	-139	142	4	121	-134	280	-127	0	149	-18	-125	12	272
status	rest	rest	rest	rest	rest	rest	step	rest	rest	step	rest	rest	rest	step
Tip of protrusion v [nm/min]	0	0	124	0	0	675	212	1175	505	482	639	70	216	-388
status	rest	rest	rest	rest	rest	step	step	step	step	step	step	rest	rest	rest
**Combined status**	**rest**						**1-2 steps**		**rest**	**1 step**	**rest**			**1 step**

PART G – COMPLEMENTARY MODEL INCORPORATING VISCOELASTICITY IN FORCE DECAY ANALYSIS

For the complementary model, forces (F) were first investigated as they are and, then, F normalized by G'_i. Both combinations were then studied together with protrusion-tip distance (R) values as they are and, R scaled by $\tan(\delta)$, and finally, R scaled by G''_i (Tables S10–S11).

Table S10. Viscoelasticity-incorporating force decay models for the data over Δt=15 min. The lowest SD/mean values of the intercept and slope are highlighted in bold (cell count N=4).

Force (F) normalized by	Distance (R) scaled by	SD/mean of intercept [%]	SD/mean of slope [%]
unnormalized	unnormalized	4	30
unnormalized	tan(δ)	10	30
**unnormalized**	**G''**	**3.3**	**30**
G'	unnormalized	10	30
G'	tan(δ)	24	30
G'	G''	4	30

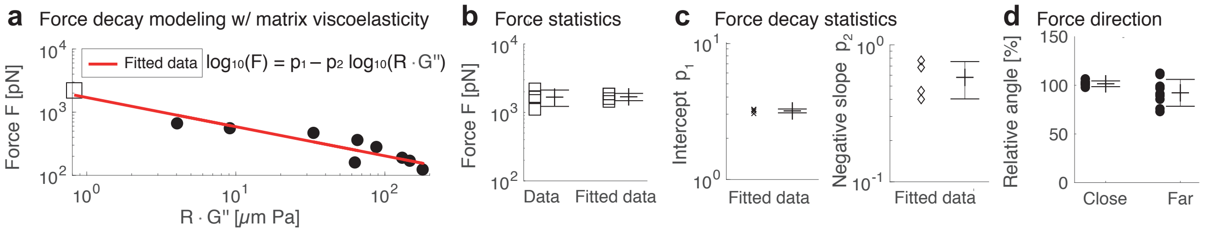

Figure S13. Force decay over Δt=15 min further analyzed. **(a)** Loss modulus-incorporating trend of force decay for one cell, shown as solid red line (*Pr<0.05, Pearson's test, datapoints n=7–10/cell, the same significance level for all cells, cell count N=4).
(b) Data for cell-exerted forces at the protrusion tip. This data and fitted data match (n.s. Pr>0.05, two-sided unpaired t-test, datapoints n=7–10/cell, cell count N=4).
(**c**) Force decay outward of the protrusion tip was consistent for the different cells based on the low scatter of the intercept (SD/mean < 3.3%) and slope (SD/mean < 30%) values for the fit lines (N=4).
(**d**) Relative angle is the angle of force-caused matrix displacement normalized by the angle of protrusion growth. "Close" and "Far" denote for 1.0 – 8.0 μm and >30 μm, respectively (N=4).

Table S11. Viscoelasticity-incorporating force decay models for the data over Δt=6.5–60 s. The lowest SD/mean values of the intercept and slope are highlighted in bold (cell count N=7).

Force (F) normalized by	Distance (R) scaled by	Max. SD/mean of intercept [%]	Max. SD/mean of slope [%]
unnormalized	unnormalized	8	48
unnormalized	tan(δ)	8	45
**unnormalized**	**G''**	**8**	**38**
G'	unnormalized	18	48
G'	tan(δ)	18	45
G'	G''	18	40

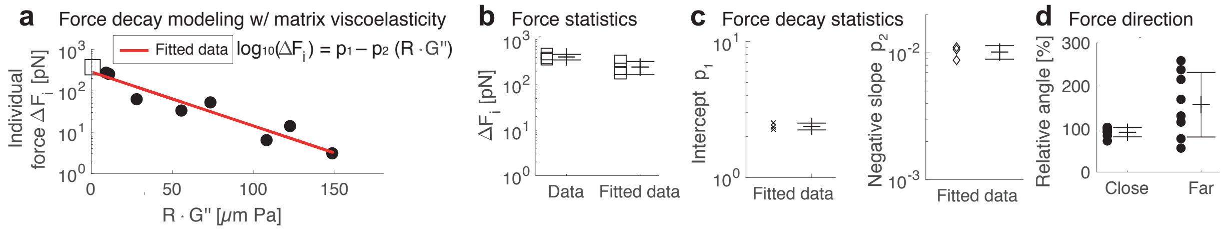

Figure S14. Force decay over Δt=1 min further analyzed. **(a)** Loss modulus-incorporating trend of force decay for one cell, shown as solid red line (*Pr<0.05, Pearson's test, datapoints n=7–10/cell, the same significance level for all cells, cell count N=4).

(b) Data for cell-exerted forces at the protrusion tip. This data and fitted data match (n.s. Pr>0.05, two-sided unpaired t-test, datapoints n=7–10/cell, cell count N=4).

(c) Force decay outward of the protrusion tip was consistent for the different cells based on the low scatter of the intercept (SD/mean < 6%) and slope (SD/mean < 38%) values for the fit lines (N=4).

(d) Relative angle is the angle of force-caused matrix displacement normalized by the angle of protrusion growth. "Close" and "Far" denote for 1.0 – 8.0 μm and >30 μm, respectively (N=4).

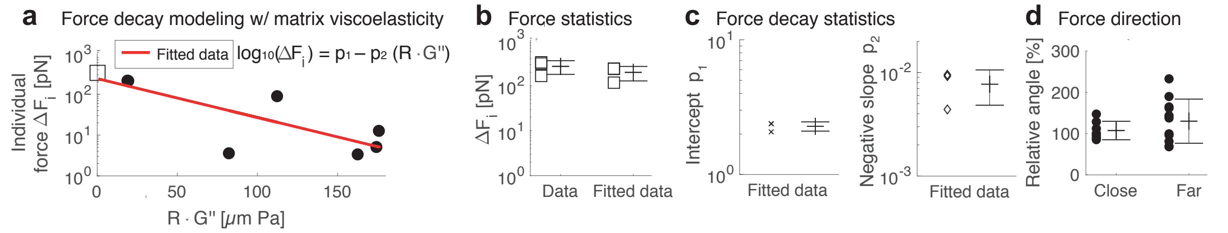

Figure S15. Force decay over Δt=6.5–10 s further analyzed. **(a)** Loss modulus-incorporating trend of force decay for one cell, shown as solid red line (*Pr<0.05, Pearson's test, datapoints n=7–10/cell, the same significance level for all cells, cell count N=3).

(b) Data for cell-exerted forces at the protrusion tip. This data and fitted data match (n.s. Pr>0.05, two-sided unpaired t-test, datapoints n=7–10/cell, cell count N=3).

(c) Force decay outward of the protrusion tip was consistent for the different cells based on the low scatter of the intercept (SD/mean < 8%) and slope (SD/mean < 37%) values for the fit lines (N=3).

(d) Relative angle is the angle of force-caused matrix displacement normalized by the angle of protrusion growth. "Close" and "Far" denote for 1.0 – 8.0 μm and >30 μm, respectively (N=3).

[1] K. M. Wisdom *et al.*, “Matrix mechanical plasticity regulates cancer cell migration through confining microenvironments,” *Nat. Commun.*, vol. 9, no. 4144, p. 1, 2018.

[2] J. Pokki, I. Zisi, E. Schulman, D. Indana, and O. Chaudhuri, “Magnetic probe-based microrheology reveals local softening and stiffening of 3D collagen matrices by fibroblasts,” *Biomed. Microdevices*, vol. 23, no. 2, p. 27, 2021, doi: 10.1007/s10544-021-00547-2.

[3] O. Chaudhuri *et al.*, “Extracellular matrix stiffness and composition jointly regulate the induction of malignant phenotypes in mammary epithelium,” *Nat. Mater.*, vol. 13, pp. 970–978, 2014.

[4] Y. Qu *et al.*, “Evaluation of MCF10A as a reliable model for normal human mammary epithelial cells,” *PLoS One*, vol. 10, no. 7, p. e0131285, 2015.

[5] S. Nam, K. Hu, M. Butte, and O. Chaudhuri, “Strain-enhanced stress relaxation impacts nonlinear elasticity in collagen gels,” *PNAS*, vol. 113, no. 20, pp. 5492–5497, 2016.

[6] S. Nam, J. Lee, D. G. Brownfield, and O. Chaudhuri, “Viscoplasticity enables mechanical remodeling of matrix by cells,” *Biophys. J.*, vol. 111, no. 10, pp. 2296–2308, 2016.
